# Age-related gene expression signatures from limb skeletal muscles and the diaphragm in mice and rats reveal common and species-specific changes

**DOI:** 10.1186/s13395-023-00321-3

**Published:** 2023-07-12

**Authors:** Tea Shavlakadze, Kun Xiong, Shawn Mishra, Corissa McEwen, Abhilash Gadi, Matthew Wakai, Hunter Salmon, Michael J. Stec, Nicole Negron, Min Ni, Yi Wei, Gurinder S. Atwal, Yu Bai, David J. Glass

**Affiliations:** Regeneron Pharmaceuticals, 777 Old Saw Mill River Road, Tarrytown, NY 10591 USA

**Keywords:** Sarcopenia, Skeletal muscle atrophy, Frailty, RNA-seq, Aging, Aging gene signature, Mitochondria, Inflammation

## Abstract

**Background:**

As a result of aging, skeletal muscle undergoes atrophy and a decrease in function. This age-related skeletal muscle weakness is known as “sarcopenia”. Sarcopenia is part of the frailty observed in humans. In order to discover treatments for sarcopenia, it is necessary to determine appropriate preclinical models and the genes and signaling pathways that change with age in these models.

**Methods and results:**

To understand the changes in gene expression that occur as a result of aging in skeletal muscles, we generated a multi-time-point gene expression signature throughout the lifespan of mice and rats, as these are the most commonly used species in preclinical research and intervention testing. Gastrocnemius, tibialis anterior, soleus, and diaphragm muscles from male and female C57Bl/6J mice and male Sprague Dawley rats were analyzed at ages 6, 12, 18, 21, 24, and 27 months, plus an additional 9-month group was used for rats. More age-related genes were identified in rat skeletal muscles compared with mice; this was consistent with the finding that rat muscles undergo more robust age-related decline in mass. In both species, pathways associated with innate immunity and inflammation linearly increased with age. Pathways linked with extracellular matrix remodeling were also universally downregulated. Interestingly, late downregulated pathways were exclusively found in the rat limb muscles and these were linked to metabolism and mitochondrial respiration; this was not seen in the mouse.

**Conclusions:**

This extensive, side-by-side transcriptomic profiling shows that the skeletal muscle in rats is impacted more by aging compared with mice, and the pattern of decline in the rat may be more representative of the human. The observed changes point to potential therapeutic interventions to avoid age-related decline in skeletal muscle function.

**Supplementary Information:**

The online version contains supplementary material available at 10.1186/s13395-023-00321-3.

## Background

Sarcopenia, the age-related loss of skeletal muscle mass and function, is a major clinical challenge for older people, associated with major co-morbidities such as osteoporosis, obesity, and type 2 diabetes [1, 2]. Sarcopenia results in a decrease in mobility, leading to frailty, falls and bone fractures, and an inability to perform daily tasks or enjoy physical activities. Not surprisingly, sarcopenia is strongly associated with mortality [3]—the decrease in walking speed which can result from sarcopenia and frailty is one of the strongest predictors of mortality in humans [4]. More recently, it became evident that muscle mass and strength were predictors of length of hospital stay in patients with moderate to severe COVID-19 [5].

Research in the mechanisms of aging and sarcopenia largely relies on the use of preclinical rodent models. These provide opportunities for intensive experimentation to define molecular mechanisms of sarcopenia, and to test potential interventions to slow down or reverse this debilitating condition. To identify molecular pathways of muscle aging and sarcopenia, we have previously used Sprague Dawley rats and conducted molecular profiling of aging by examining gene expression changes in multiple tissues, including the gastrocnemius muscle, throughout multiple time points in the lifespan of the animal [6, 7]. These studies revealed gene pathways that are globally regulated by aging, as well as gene pathways associated with sarcopenia in the rat gastrocnemius muscle [6, 7].

The aim of the present study was to examine multiple skeletal muscles at multiple times in two commonly used preclinical species, mice and rats. Thus, we studied gene expression changes with age throughout the animals' lifespan (at 6, 9, 12, 18, 21, 24, and 27 months) in the diaphragm, gastrocnemius, soleus, and tibialis anterior muscles. We analyzed tissues from inbred male and female C57Bl/6J mice and outbred male Sprague Dawley rats.

Here, we applied a previously established method that allows for identification of multi-tissue and multi-time point aging signatures [7]. We discovered similarities and differences between mouse and rat muscle aging. We identified genes and gene pathways that are regulated in the same direction in multiple muscles, giving some indication of common mechanisms of aging. We also observed more dramatic age-related gene expression changes in rats compared with mice, and this finding was consistent with a more pronounced loss of muscle mass in aged rats compared with mice. It is our hope that this dataset will serve as a valuable resource for further molecular insights into mechanisms of skeletal muscle aging and sarcopenia.

## Methods

### Animal maintenance and tissue collection

All procedures involving animals were approved by the Institutional Animal Care and Use Committee of Regeneron and New York Medical College (NY, USA). Male and female C57BL/6 J mice were purchased from Jackson Laboratory (USA) at various ages and maintained at Regeneron animal holding facilities for ~12 weeks prior to tissue collection under specific pathogen free (SPF) conditions. Mice were housed in groups of 5 per cage with controlled temperature and light (22℃, 12-h light/12-h dark cycle: lights on at 0600 h/lights off at 1800 h) and with ad libitum access to food (PicoLab Rodent Diet 20, Lab Supply) and water. Male Sprague Dawley (SD) rats were purchased at 3–4 weeks of age from Envigo (Indianapolis, USA) and aged at Envigo under specific pathogen free (SPF) conditions. Rats were imported to New York Medical College animal holding facilities 8–12 weeks prior to tissue collection. Once imported, rats were housed two per cage at the SPF facility with controlled temperature and light (22℃, 12-h light/12-h dark cycle: lights on at 0600 h/lights off at 1800 h) and with ad libitum access to food (2014 Teklad Global 14% Protein diet, Envigo) and water. Tissue collection occurred over several months and tissues from the same age group (*n* = 8–12 per group for most groups, but with one group containing 13 animals) were collected on the same day between 9am and 1 pm. Prior to tissue collection, mice were killed by CO_2_ asphyxiation. Rats were anesthetized with 3.5% isoflurane and killed by exsanguination and thoracotomy. Diaphragm, gastrocnemius, soleus, and tibialis anterior muscles were preserved in RNA later and stored at −80℃.

### RNA sequencing

RNA was extracted from tissues preserved in RNA later using MagMax-96 total RNA isolation kit (Thermo Fisher). The RNA concentration was quantified using NanoDrop Spectrophotometer (NanoDrop Technologies), and the integrity was assessed using Fragment Analyzer (Agilent). Strand-specific total RNA-seq libraries were prepared using Kapa RNA HyperPrep kit (Roche) with Fastselect rRNA HMR ribosome RNA removal kit (Qiagen). RNA-seq libraries were sequenced at 100 million reads using Illumina NovoSeq 6000 platforms in paired-end mode to a length of 2 × 76 bp base-pairs. Sequencing reads were processed with ArrayStudio RNA-seq pipeline to quantify gene expression levels. For mouse, we used 10 mm genome reference and Refseq based in house gene model. For rat, we used B6.0 genome reference and Refseq based in house gene model.

### Identification of age-related genes

**Fig. 1:**
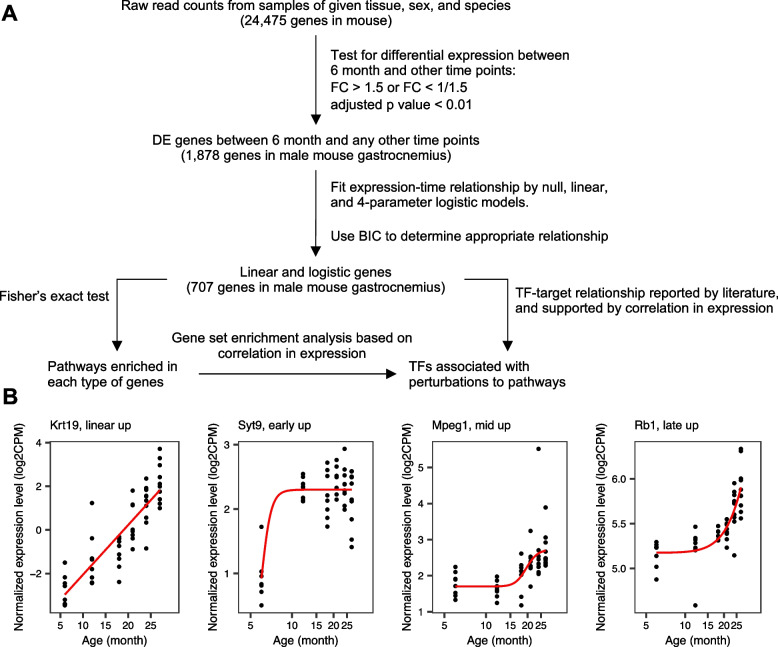
Summary of the analysis. **A** Flowchart for identifying age-related genes, pathway enrichment analysis, and transcription factor (TF) association analysis. RNA-seq data were processed from each species, sex, and tissue separately. The raw gene expression counts were normalized using the limma package, and the normalized data were used to first identify differentially expressed (DE) genes. Genes were considered differentially expressed if their expression was upregulated or downregulated by ≥ 1.5 fold at any age compared to 6 months, with the adjusted *p* value of > 0.05. To define age-related genes, we fitted the trend of a DE gene by null, linear, and a 4-parameter logistic model. Bayesian information content (BIC) was used to identify the best model among the three, and DE genes with a linear or a logistic trend were considered age-related. Next, age-related genes, with linear and logistic behaviors were used to perform pathway enrichment analyses and transcription factor (TF) motif enrichment. **B** Examples of age-related genes that follow a linear pattern, or early, mid-, or late-logistic patterns (shown for the mouse gastrocnemius)

We identified age-related genes from RNA-seq data by using the earlier described method [7], with minor modifications (Fig. 1A). We processed the data from each species, sex, and tissue separately. We used the limma package [8] to normalize the raw gene expression counts and identify differentially expressed (DE) genes between 6 month and any other age. Genes were considered DE if their expression was upregulated or downregulated by >1.5 fold at any age compared to 6 months, and the Bonferroni adjusted *p* value < 0.05.

Next, for each differentially-expressed gene, we fitted a null model, a linear model, and a 4-parameter logistic model to the log2(CPM) over time. We used the Bayesian Information Content (BIC) of each model to identify the best model, which should have the smallest BIC. We focused on genes for which the linear_BIC or the logistic_BIC was smaller than the null_BIC by more than 6, which is a relatively stringent cutoff [9], in order to capture a strong monotonic increase or decrease in gene expression with age. These genes were further classified as linear, unless the logistic_BIC was smaller than the linear_BIC by more than 2. This gives the simpler linear relationship higher priority than the more complicated logistic relationship. This less stringent cutoff was sufficient to separate apparent logistic genes from the linear ones (Fig. 1B), while we note that a clear distinction between the two classes was not always present due to the sample size. We also note that, in rare cases, a difference of about 4 in BIC score is sufficient to classify a gene as linear over the null model (e.g., null_BIC = 100, linear_BIC = 95.9, logistic_BIC = 93.9). Nevertheless, because both the linear_BIC and logistic_BIC are smaller than the null_BIC by 4 or more, there is enough evidence to reject the null hypothesis that the given gene’s expression is independent of age. Using a more stringent BIC cutoff does not greatly reduce the numbers of age-related genes (Fig. S1). Genes that were classified as logistic were further classified as early-logistic if the inflection point is smaller than 12 months, mid-logistic if between 12 and 21 months, and late-logistic after 21 months. Linear genes with positive slope and logistics genes with positive Hill coefficient are classified as increasing with age and decreasing with age otherwise.

### Pathway enrichment analysis

We downloaded species-specific Hallmark pathways [10] and REACTOME pathways [11] and their associated genes from MSigDB [12]. Genes that are not present in our gene annotations were removed. We discarded pathways that overlap with less than 5 genes without performing Fisher’s exact test. The *p* values were adjusted with a Bonferroni correction for multiple hypothesis testing, and an adjusted *p* value smaller than 0.01 was considered significant overlap between pathway and age-related genes. To further remove potential false hits, the downstream analysis used only pathways that were enriched in two tissues of the same or different species.

### Comparing mouse and rat orthologs

We used gprofiler2 [13] to identify orthologous genes between rat and mouse. We kept the first rat (mouse) ortholog candidate of each mouse (rat) gene returned by the program. Still, because one rat gene can be orthologous to multiple mouse genes (and vice versa), the number (15,108) of rat genes that have a mouse ortholog does not equal to the number (14,838) of mouse genes that have a rat ortholog.

We performed Fisher’s exact test to determine whether the perturbation of orthologous age-related genes statistically significantly share the same direction across the two species. For example, in mouse diaphragm, there are 82 upregulated genes that have a rat ortholog, and for 20 of them, the corresponding rat orthologs are also upregulated in rat diaphragm. The number of mouse genes that have a rat ortholog is 14,838, among which the rat orthologs of 369 are age-related genes that increase expression with age in diaphragm. Given these numbers, the probability of observing the 20 cases or more is $7.5{E}^{-15}=\sum_{i=20}^{82}\frac{\left(\genfrac{}{}{0pt}{}{369}{i}\right)\left(\genfrac{}{}{0pt}{}{14469}{82-i}\right)}{\left(\genfrac{}{}{0pt}{}{14838}{82}\right)}$.

### Identifying transcription factors (TFs) associated with age-related pathways

We compiled TF-gene interactions in human and mouse from ChEA3 [14], TRRUST version 2 [15], and hTFtarget [16]. The interactions were filtered separately for rat and mouse, by requiring that the TFs were from the GO ontology terms GO0003700, GO0003712, and GO0140297 [17, 18] of the given species and that both genes of an interaction are age-related genes in at least one tissue of the given species. The final lists contain 235 TFs and 92.1 k interactions in rat and 79 TFs and 11.9 k interactions in mouse.

To associate TFs with a pathway, we searched for TFs whose target genes are enriched within the pathway. Because many of the pathways are biologically related, we instead checked the level-2 REACTOME systems, which is a superset of related pathways. For example, “Signaling by Interleukins” is enriched in the linear-up genes in multiple rat tissues. This pathway belongs to the level-2 REACTOME term “Cytokine Signaling in Immune System”, which is a subset of the top-level system “Immune System”. We identified linear-up genes that are shared by two or more rat tissues and belong to “Cytokine Signaling in Immune System”.

For each enriched level-2 system, we calculated the Spearman correlation in expression levels between the relevant age-related genes and the interacting TFs across all samples of the given species. We built on the idea of Gene Set Enrichment Analysis [12] that ranks genes by fold-change in expression and identifies pathways whose genes have significantly extreme rankings. In our case, we ranked all interactions between TFs and age-related genes (observed in at least one tissue) by the Spearman correlation. A “pathway” is the interactions between a TF and the age-related genes in a level-2 system described above. “Pathways” with extreme rankings were identified by using the R package fgsea (fast pre-ranked gene set enrichment analysis http://biorxiv.org/content/early/2016/06/20/060012), requiring the adjusted *p* value < 0.05 and ignoring TFs with less than 5 target genes.

### Reverse transcription quantitative PCR (RT-qPCR)

RNA samples from mouse and rat muscles were reverse transcribed to cDNA using SuperScript IV VILO kit (Thermofisher Scientific). The cDNA was then used for RT-qPCR to confirm findings from RNA-seq analyses, as well as to determine the expression profile of selected genes. For each muscle, the transcript levels were normalized to the geometric mean of 2–4 reference genes. Reference genes were determined based on RNA-seq results and further validated by RT-qPCR. Tables S3 and S4 list specific probes and primers used for mouse and rat muscles.

## Results

### Phenotypes of skeletal muscles in mice and rats across multiple ages

To determine if mice and rats undergo sarcopenia—the age-related decline in skeletal muscle mass, resulting in weakness and frailty—we first compared hindlimb muscle (gastrocnemius, tibialis anterior and soleus) weights across a range of ages in male and female C57Bl6J mice, and, separately, in male SD rats (Fig. S2). In male and female mice, weights for gastrocnemius, tibialis anterior, and soleus remained largely unchanged from 6 to 21 months (Fig. S2A and B). There was an age-related decrease in the weights of these muscles at 24 months and no further change by 27 months (Fig. S2A and B). Weights of the gastrocnemius, the tibialis anterior, and the soleus muscles in 24-month male mice were ~17%,  ~15%, and ~26% lower compared with 6 months (Fig. S2A). In female mice, weights of the gastrocnemius and the tibialis anterior muscles were ~13% and ~15% lower at 24 months compared with 6 months (Fig. S2B). In female mice, the soleus muscle weights were comparable across all analyzed ages (Fig. S2B). Age-related loss of hindlimb muscle mass was more severe in rats compared with mice (Fig. S2C). In rats, the gastrocnemius and the tibialis anterior muscles gradually and continuously lost weight from 18 to 27 months. At 27 months, the gastrocnemius and the tibialis anterior muscles weighed 45% and 40% less compared to 9 months (Fig. S2C). The weight of the rat soleus muscle decreased by 26% between 9 and 24 months, with no further change by 27 months (Fig. S2C).

### Identification of age-related genes in the limb and the diaphragm muscles in mice and rats

To identify age-regulated genes in rodent skeletal muscles, we performed gene expression profiling by RNA sequencing (RNA-seq) of diaphragm, gastrocnemius, soleus, and tibialis anterior from male and female C57BL/6 J mice and male SD rats. We used rodents aged 6, 12, 18, 21, 24, and 27 months, and in the case of rats, an additional 9-month group was also included. For mice, we analyzed 6–10 animals for each age group, for each sex. For rats, 9–13 animals for each age group were analyzed. A detailed sample list is provided in Table S1. The gene expression data are available at the NIH GEO under GSE226117 and GSE226118. In the main text, we included data from male mice and male rats. Comparisons between male and female mice are included in the supplemental files. To confirm RNA-seq analyses, we performed RT-qPCR amplification of top five up- and downregulated genes in all muscle groups (Figs. S10 and S11).

We followed the analysis strategy used in our earlier study [7]. First, we identified age-regulated genes that met the following criteria: differentially expressed between 6 month and any other age, with a fold change of >1.5 (in either direction) and the adjusted *p* value < 0.01 (Fig. 1A). Next, we filtered the differentially expressed genes (DEGs) to retain genes whose expression monotonically increased or decreased with age, and divided these genes into two broad classes: linear and logistic (Fig. 1A). “Linear” genes were age-related genes whose expression increased or decreased at a constant pace across the analyzed ages (Fig. 1B). Expression of the “logistic" genes changed at a set age (the inflection point), thereon remaining at a new set level. Depending on when the inflection point took place, the logistic genes were further classified into early-logistic, mid-logistic, and late-logistic (Fig. 1B). Table S2 contains all age-related genes in searchable Excel format with log2-fold changes relative to 6 months and respective adjusted *p* values.

### More age-related genes in rat skeletal muscles compared with mice

**Fig. 2:**
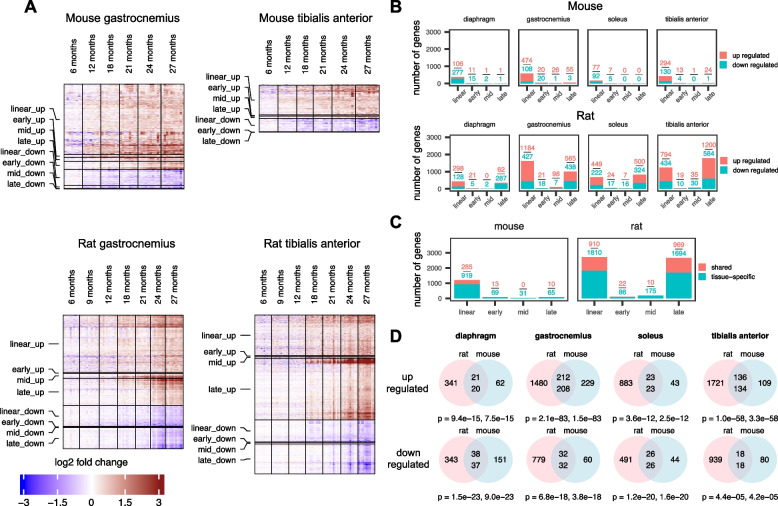
More age-related genes were demonstrated in the skeletal muscle of rats compared with mice. **A** Heatmaps showing expression of age-related genes in the gastrocnemius and the tibialis anterior muscle in male mice and male rats across multiple ages (6–27 months). Gene expression fold change was calculated using the average normalized log2 CPM (counts per million) of samples at 6 months as reference. Red color represents increase in gene expression relative to 6 months, with the deepest red being an increase of ≥ eight fold. Blue color represents decrease in gene expression relative to 6 months, with the deepest blue being a decrease of ≥ eight fold. In heatmaps, each row represents a single gene, and each column represents a single animal. *N* = 6–8 mice and *N* = 9–13 rats per group respectively. **B** Numbers of linear and logistic age-related genes in diaphragm, gastrocnemius, soleus, and tibialis anterior muscles from male mice and rats. Red boxes represent number of upregulated genes, and green boxes represent number of downregulated genes. **C** Numbers of age-related genes whose expression changed in the same direction (up or down) and had the same classification (linear or logistic) in at least two muscles from the same species (mouse or rat). Red boxes represent upregulated genes and green boxes represent downregulated genes. **D** Venn diagrams showing age-related genes that were orthologous in the muscles of rats and mice. Pink circles are rat age-related genes that have a mouse ortholog, and blue circles are mouse age-related genes that have a rat ortholog. Top numbers in the intersections show the number of rat genes whose expression changes in the same direction as their respective mouse ortholog (significance shown by the first *P* value). Bottom numbers in the intersections show the number of mouse genes whose expression changes in the same direction as their respective rat ortholog (significance shown by the second *P* value). *P* values are derived by Fisher’s exact test (see the “Methods” section for details)

Rat skeletal muscles experienced more age-related genes (on average ~ 2000 genes per muscle) compared to mice (on average ~ 300 genes per muscle) (Fig. 2A, B), and the magnitude of the fold change in age-related gene expression in rats was larger compared with that in mice (Fig. 2A). As a precaution, to test whether the larger number of age-related genes found in rat skeletal muscles was due to the larger sample size (*N* = 9–13 for rats versus *N* = 6–10 for mice. Also, see Table S1), we randomly down-sized sample numbers in each age group in rats to match sample numbers in mice. Still, the number of age-related genes found in rat muscles remained largely unchanged (Fig. S3). In the muscles of both species, the majority of age-related genes were up-regulated genes (Fig. 2B). Mouse skeletal muscles mostly demonstrated linear age-related genes (Fig. 2B). In rat skeletal muscles, we predominantly found linear and late-logistic genes (Fig. 2B). Early- and mid-logistic genes were rare in all rodent muscles (Fig. 2B). These results suggest that the lower limb and the diaphragm muscles in rats show more robust consequences of aging, compared with mice, and these differences are particularly pronounced at later ages (≥ 21 months) (Fig. 2B).

We also performed comparisons among muscle types within each species. In both species, the gastrocnemius and the tibialis anterior muscles had higher numbers of age-related genes compared with the diaphragm and the soleus muscles (Fig. 2B). These results may reflect differences in the myofiber type composition (e.g., fast versus slow), the functional load for each muscle type and the origin of innervation for each muscle (e.g., innervation by the lumbar versus cervical motoneurons). The proportion of age-related genes shared by two or more muscle types is ~ 34% in rats and ~ 22% in mice (Fig. 2C).

To further investigate the similarities and differences in skeletal muscle aging gene signatures from mice and rats, we identified orthologous age-related genes for each muscle for each species (Fig. 2D). Interestingly, expression of orthologous age-related genes from mice and rats generally changed in the same direction (Fig. 2D). In other words, orthologous age-related genes that were upregulated in mouse muscles also tended to be upregulated in rats and vice versa, and orthologous age-related genes that were downregulated in one species showed similar behaviors in the other species (Fig. 2D).

The skeletal muscles from female mice showed 231 more age-related genes in total compared with males (Fig. S4A). Similar to males, the majority of age-related genes in female muscles were linearly upregulated (Fig. S4A). Figure S4B shows the total numbers of upregulated and downregulated age-related genes for male and female muscles and the overlap between the species.

### Elevated inflammatory and immune responses are common in aging rodent skeletal muscles

To understand biological functions of age-relate genes with linear, early-, mid-, and late-logistic behavior, we performed gene pathway enrichment using REACTOME and Hallmark databases. We focused on pathways enriched in two or more muscles in each species. This approach removed a few pathways enriched with early- and mid-logistic genes that appear in only one tissue (Fig. 2B).

**Fig. 3:**
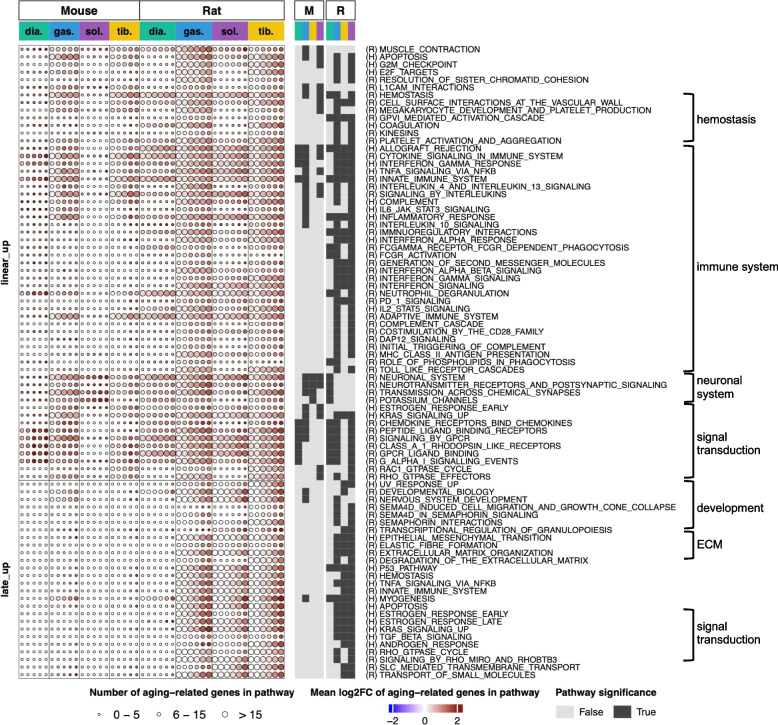
Graphic representation of pathways enriched by upregulated age-related genes in diaphragm, gastrocnemius, soleus, and tibialis anterior muscles from male mice and rats across multiple ages (from 6 to 27 months). Pathways enriched by linear-up and late-up genes in each muscle (from mice and rats, respectively) are depicted graphically as circles. Each column of circles, for each muscle, corresponds to the comparison between 6 month and the older age (12, 18, 21, 24, and 27 months for mice and 9, 12, 18, 21, 24, and 27 months for rats). The circle size represents the number of age-related genes enriched to each pathway: small circles denote enrichment of the pathway by 0–5 genes; medium circles, enrichment by 6–15 genes and large circles, enrichment by > 15 genes. The circle color indicates the average fold-change in the expression levels of these genes versus 6 months, with the deepest red being an increase of ≥ four fold. Dark squares on the right-hand side of the graphical heatmap indicate that enrichment for a specific pathway was statistically significant

Pathways upregulated with aging were mainly enriched by linear and late logistic age-related genes, and there were more such pathways in rat muscles compared with mice (Fig. 3). Linear upregulated pathways were found in all four muscles in both species; however, the rat muscles were more affected compared with the mouse muscles (Fig. 3). To be cautious, we decided to test if these differences could have been a result of fewer age-related gene perturbations between young and old mice, and/or merely a difference between the gene and pathway annotations for mouse and rat. However, when we repeated the analysis by using a lenient set of age-related genes and a common annotation for genes and pathways, rat muscles still showed larger perturbations (Fig. S5), suggesting a genuine difference in the age-related gene perturbations between species.

In addition, there were muscle type specific differences within species: In mice, the gastrocnemius and the tibialis anterior muscles were more affected compared with the diaphragm and the soleus. In rats, the diaphragm muscle had the least number of age-related gene pathways (Fig. 3).

In both species, pathways related to inflammation, cytokine signaling, and immune response were prevalent and were linearly upregulated in at least three muscle types (Fig. 3). Figure S6A exemplifies individual age-related genes enriched to the immune theme, while Table S2 includes expression values of all age-related genes. Again, the soleus was the least affected muscle in mice and the diaphragm was the least affected muscle in rats. Taken together, these results suggest that there is a gradual increase in immune and inflammatory responses in rodent skeletal muscles (both mice and rats) during aging. Among other linear-up pathways, shared by both species and conspicuous in at least two muscles, were apoptosis, hemostasis, chemokine signaling, KRAS (Kirsten rat sarcoma virus), and GPCR (G protein-coupled receptor) signaling.

In addition, we searched for transcription factors (TF) that may have been potential regulators of linearly upregulated pathways shared between mice and rats. Briefly, we pooled data on potential interactions between TFs and age-related genes from public databases and identified TFs whose target genes are enriched in a pathway (see the “Methods” section for details). A few TFs associated with the linear-up pathways were shared by both species: e.g., Runx1, Runx3, Ikzf1, and Bcl3 (Fig. S9A and B).

Pathways enriched by late-upregulated genes (late-up pathways) were mostly observed in rat skeletal muscles (Fig. 3), which is in agreement with the finding that there were many more late-logistic genes in rats compared with mice (Fig. 2B). Within the rat species, the diaphragm had the least number of late-up pathways (Fig. 3). In mice, the gastrocnemius was the only muscle where we saw the late-up pathways (Fig. 3). Interestingly, late-up pathways termed “myogenesis”, “developmental biology”, and “nervous system development” were observed in all rat muscles and in the mouse gastrocnemius muscle (Fig. 3). Closer examination of individual genes enriched to these pathways point to age-related deterioration of the neuromuscular junctions (NMJs) and functional denervation of myofibers in old muscles. A group of these genes is shown in Fig. 5A and discussed below.

In all lower limb muscles in rats, we observed pathways enriched by late-up genes that were related to extracellular matrix organization, TNF alpha and TGF beta signaling, estrogen response (Fig. 3). In the skeletal muscles from female mice, upregulated age-related genes enrich to similar pathways as in males (Fig. S7). Similar to males, the linear-up pathways are prevalent in female gastrocnemius and tibialis anterior muscles and are related to immune response, cytokine signaling and inflammation, neuronal system regulation, and signal transduction (Fig. S7). In addition, similar to males, late-up pathways were seen predominantly in the gastrocnemius muscle in females and were related to myogenesis and development (Fig. S7).

### Metabolism and mitochondrial functions are reduced in rat skeletal muscles

**Fig. 4:**
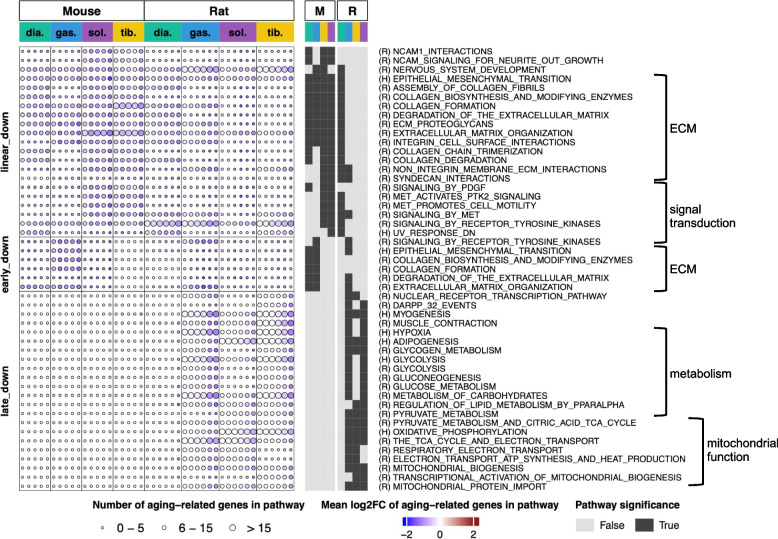
Metabolism and mitochondrial functions are reduced in rat skeletal muscles, but not in mouse skeletal muscles. Graphic representation of pathways enriched by downregulated age-related genes in diaphragm, gastrocnemius, soleus, and tibialis anterior muscles from male mice and rats across multiple ages (from 6 to 27 months). Pathways enriched by linear-down, early-down and late-down genes in each muscle (from mice and rats respectively) are depicted graphically as circles. Each column of circles, for each muscle, corresponds to the comparison between 6 month and the older age (12, 18, 21, 24, and 27 months for mice and 9, 12, 18, 21, 24, and 27 months for rats). The circle size represents the number of age-related genes enriched to each pathway: small circles denote enrichment of the pathway by 0–5 genes; medium circles, enrichment by 6–15 genes and large circles, enrichment by > 15 genes. The circle color key indicates the average fold-change in the expression levels of these genes versus 6 months, with the deepest blue being a decrease of ≥ four fold. Dark squares on the right-hand side of the graphical heatmap indicate that enrichment for a specific pathway was statistically significant

In the skeletal muscles from both species, gene pathways related to the extracellular matrix (ECM) organization were downregulated linearly throughout life and, to a lesser extent, early in life (Fig. 4). In mice, age-related downregulation of the ECM pathways was seen in all four muscles, while in rats, predominately in the diaphragm (Fig. 4).

A striking difference between mouse and rat muscles emerged from comparing pathways downregulated late in life (Fig. 4). These pathways were exclusively found in the lower limb muscles of rats and enriched by metabolic and mitochondrial genes (Fig. 4 and Fig. S6B). Interestingly, downregulation of metabolic and mitochondrial pathways, at least at the gene expression level, was not seen in the rat diaphragm muscle (Fig. 4). These differences between mouse and rat muscles remained true even after we repeated the analysis using a lenient set of age-related genes and a common gene and pathway annotations (Fig. S8). Thus, unlike rats, mouse skeletal muscles show no apparent age-related reduction in gene pathways linked with “metabolism” or “mitochondrial functions”.

Since gene pathways linked with the “mitochondrial function” are downregulated in the skeletal muscles of old rats, we investigated the transcription factors associated with these pathways (Fig. S9A). Expression of two transcription factors, Esrra and Nfyb, shows a positive correlation with genes enriched to mitochondrial pathways (Fig. S9A): these genes have been shown to be vital to mitochondrial health [19, 20]. We found that Esrra is a late-down gene in rat gastrocnemius, soleus, and tibialis anterior muscles and Nfyb is a late-down gene in the rat tibialis anterior (Fig. S13). This finding suggests that Esrra and Nfyb may promote downregulation of mitochondrial pathways in the skeletal muscles from old rats.

In female mice, downregulated age-related genes enrich to similar pathways as seen in males (Fig. S7). Similar to males, we did not observe late-life downregulation of the metabolic and mitochondrial pathways in the skeletal muscle of female mice (Fig. S7).

### Age-related expression of genes related to skeletal muscle denervation and atrophy

**Fig. 5:**
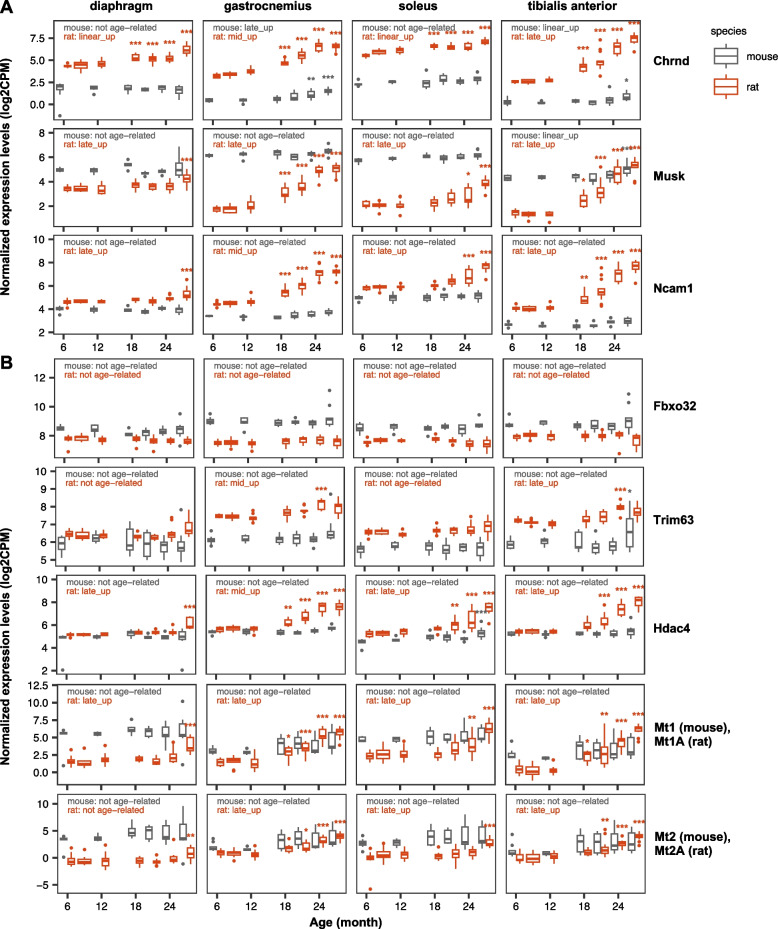
Expression levels of selected genes in the muscles of male mice and rats. **A** Common marker genes of myofiber denervation. **B** Common marker gene of skeletal muscle atrophy. Boxplots show normalized expression levels of these genes from 6 to 27 months, with “outliers” marked as dots. Asterisks indicate significant difference compared to 6 months. Bonferroni adjusted *p* value * < 0.05, ** < 0.01, and *** < 0.001. See Table S2 for the exact fold changes and *p* values for these genes. Genes that are not differentially expressed at any age compared with 6 months or do not have linear or logistic trends are labeled as “not age-related”. For age-related genes, the behavior patterns are indicated: e.g., “late-up”

In addition to conducting unbiased gene pathway analyses, we surveyed specific genes that have been previously shown to be upregulated in settings of skeletal muscle denervation atrophy [21–24]. Gene markers of myofiber denervation, such as Chrnd (acetylcholine receptor delta subunit), MuSK (muscle specific kinase), and Ncam1 (neuronal cell adhesion molecule 1) were significantly increased in old rat muscles, but not mouse muscles (Fig. 5A). One exception was the gastrocnemius muscle in mice that showed increase in Chrnd late in life (Fig. 5A). In addition to markers of myofiber denervation, we examined five genes that are associated with skeletal muscle atrophy (Fig. 5B). There was a statistically significant upregulation of MuRF1/Trim63 (Muscle RING-finger protein-1), Hdac4 (Histone deacetylase 4), Mt1/Mt1A, and Mt2/Mt2A (Metallothionein 1 and 2) transcripts in old rat muscles but not mouse muscles (Fig. 5B). This observation is consistent with much more pronounced muscle atrophy in the rat compared with the mouse (Fig. S2). MAFbx/Atrogin1/Fbxo32 was not upregulated during sarcopenia in the mouse or the rat, which is in agreement with our earlier observations [6].

## Discussion

We previously published an aging gene signature from rats which surveyed one skeletal muscle, gastrocnemius, in addition to three other tissues: liver, kidney, and the hippocampus [7]. Here, we sought to survey rodent skeletal muscle more deeply, to ask more granular questions:

1. Are there consistent mRNA expression changes between rats and mice—as one might expect, since they are both rodents?
2. Are there differences in age-related gene expression in the rat *versus* the mouse, which might suggest one might be a “better” or more representative model for what happens in the human—if, for example, one of the rodent species underwent changes which are also found in the human?
3. Are there any changes specific to the mouse or the rat muscle—i.e., not seen in the other species?
4. Do the various muscles within a species demonstrate similar or different age-related gene expression changes?
5. Might any of the age-related gene expression changes in mice or rats explain their loss of muscle mass, or the decline in the patency of the neuromuscular junction which accelerates age-related weakness, since the skeletal muscle becomes functionally denervated [25–28]?

In addition to studying multiple limb muscles, we added the diaphragm to this study, since this muscle is contracting constantly throughout life, to get an idea as to whether constant use distinguishes that muscle from any of the aging changes found in the limbs. We surveyed these gene expression changes in conjunction with age-related changes in skeletal muscle mass, which have previously been associated with declines in muscle function [6]; indeed, while some have debated whether muscle hypertrophy causes an improvement in muscle function, no one argues against the notion that decreases in muscle mass induce muscle weakness—since there are fewer sarcomeres to contract.

Before undertaking to answer these questions, we did some quality control work. First, we compared our data with publicly available gene expression datasets for rodent skeletal muscle aging from SarcoAtlas (https://sarcoatlas.scicore.unibas.ch) [29–31]). SarcoAtlas is a valuable resource that includes time course transcriptomic data from mice (*N* = 8–9 per group) and from rats (*N* = 9–10 per group). Specifically, data from Wistar rat gastrocnemius muscles in SarcoAtlas includes five ages, with 24-month-old rats being the oldest group [30, 32]. Eighty-eight percent of age-related upregulated genes and 81% of downregulated genes identified in the Wistar rat gastrocnemius are consistent with upregulated and downregulated genes, respectively, in our study (SD rat gastrocnemius).

The mouse aging time course in SarcoAltas includes samples from the gastrocnemius muscle of six different ages, ranging from 8 to 28 months [29]. Age-related genes from SarcoAtlas overlap with our data: 24% for upregulated genes and 8% for downregulated genes are consistent with our upregulated and downregulated genes, respectively. We note that SarcoAtlas defines age-related genes differently than us: they use principal component analyses to identify genes that align well with the age of the animal [29]. While it was reassuring to see consistency between our data and prior-published studies, we should note this current study is unique in that it combines multiple elements which allow for novel findings: the multiplicity of time points studied, the number of muscles analyzed, the *N* of animals in each group analyzed to better assure data accuracy, and the number of species analyzed—which uniquely allows for mouse versus rat comparisons. Also, we analyzed both, males and females in mice.

Having gone through the relevant literature comparisons, we were ready to address the questions asked. As to the question: Are there differences between age-related changes in rat muscle versus mouse muscle, the answer is clearly “yes”, and these differences are quite striking. The rat but not the mouse showed a decrease in mitochondrial gene expression as a result of age, a finding which has been reported in human sarcopenia [33]. Also, the rat showed a more dramatic increase in innate immune and inflammatory signaling, compared with the mouse, including an increase in interferon signaling, that may be a marker for DNA damage in old muscles [34–36]. We had previously reported upregulation of interferon response genes in the gastrocnemius and the non-muscle tissues (liver, kidney, and hippocampus) in the rat [7]. Age-related increase in the innate immune and inflammatory signaling was also seen in mouse skeletal muscles; however, this response was more modest compared with rat and was primarily localized to the fast limb muscles (gastrocnemius and tibias anterior). Age-related downregulation of the extracellular matrix genes was another commonality between mouse and rat muscles, albeit this downregulation was more prevalent in mouse muscles. At least in mice, downregulation of the ECM theme occurs in multiple tissues throughout the lifespan [37]. Extracellular matrix changes have been reported to occur during aging and sarcopenia, resulting in decreased muscle function [38]; the extracellular matrix in muscle is critical for anchoring the muscle fibers, and thus the sarcomere, to allow for muscle contraction [39–41].

As to the question as to whether one rodent model be superior over the other when it comes to modelling the human, both species may be useful; however, differences need to be taken into account as these will influence what therapeutic targets are being investigated.

Overall, we found that the rat has a wider range of age-related transcriptional changes. Modeling muscle aging in the mouse may leave out some of the age-related changes that have been found to be relevant to the human sarcopenia, e.g., decline in signatures of mitochondrial function and oxidative phosphorylation [33]. We note that the lack of reduced mitochondrial function in our mouse muscle data is consistent with the findings of Ham et al. [30], while Borsch et al. [29] reported reduced mitochondrial function in the muscles of mouse, rat, and human. These discrepancies may reflect a difference in how the gene pathway enrichment tests were performed: Ham et al. 2020 and our study used Fisher’s exact test while Borsch et al. 2021 used GESA [29]. Thus, changes in mitochondrial function in mouse muscles might be relatively subtle and subjected to interpretation depending on the method used in analysis. Indeed, when we analyzed the rodent time course data from SarcoAltas, we found more genes related to mitochondrial function are reduced in rat muscles compared with mouse muscles.

In addition, it should be noted that our oldest groups of male and female mice were 27 months. Other laboratories have found more pronounced age-related muscle wasting and more robust molecular changes in mice if they are allowed to age even more, resulting in “geriatric mice” [30, 42–44]. We find it challenging to age C57Bl6J mice (purchased from the Jackson laboratory, USA) beyond 27 months, since there is a high attrition rate (> 50%) and many surviving mice develop various types of malignancies (tumors). With the high tumor burden, it is challenging to differentiate whether skeletal muscles are responding to natural aging or cachectic environment.

Substantial differences in the sarcopenic phenotype between mice and rats are intriguing. We found that lower limb muscles in rats are more impacted by aging compared to the same muscles in mice. One possibility for more pronounced sarcopenia in the lower limbs of rats compared with mice, is more severe age-related deterioration of muscle innervation. Even though myofiber denervation occurs in both mice [45, 46] and rats [32], more severe loss of innervation may be a significant contributor to the more severe sarcopenia seen in rats. To this end, we observed a more robust dysregulation of the NMJ stability markers in the lower limb muscles of rats, compared with mice, pointing to more severe functional denervation of myofibers in rats.

Interestingly, the diaphragm muscle was relatively spared, and we saw milder changes in the aging transcriptome in the rat diaphragm compared with the lower limb muscle. Similar to our findings, Pannérec et al. reported more severe sarcopenia in the lower limb muscles of old Wistar rats, compared with the triceps muscle [32]. These differences were attributed to more severe age-associated changes in the lumbar-region of the spinal cord that innervates lower limbs, compared with the cervical region that innervates the diaphragm [32]. In rats, diaphragm motor units are innervated by phrenic motoneurons located at C3–C5 levels of the cervical spinal cord [47]. Thus, it is possible that similar to sparing of the triceps muscle in old rats [32], the diaphragm muscle is also spared due to relatively robust motoneurons in the upper region of the spinal cord.

Deterioration of NMJs and myofiber denervation is also a feature of human sarcopenia [25, 27, 48]. Moreover, loss of motoneuron numbers in old human spinal cords may be a contributing factor for severe cases of sarcopenia encountered in geriatric population [25].

Thus, the presence of a more severe denervation-driven phenotype in rats, combined with the significant downregulation of mitochondrial function, should be taken into account when choosing the mouse or the rat to model human sarcopenia. The rat is likely to be a more useful model to discover targets related to loss of innervation and mitochondrial disfunction in settings of sarcopenia.

As to the key question about whether there are consistent changes, which might point to evolutionary conserved mechanisms of aging, the main alteration which is consistent is the increase in innate immune and inflammatory signaling, with a particular focus on interferon signaling. Interestingly, there was a striking overlap between the age-related pathways identified in our study in both species with the pathways upregulated in cultured senescent cells [49]. For instance, pathways enriched by genes responsive to interferon alpha and gamma, TNF-alpha, and other pro-inflammatory cytokines that are induced in old rodent muscles, are also induced in senescent cells [49]. Increase in interferon responsive genes and pro-inflammatory pathways may suggest accumulation of senescent cells and DNA damage events in old rodent muscles. DNA damage itself is capable of inducing interferon response genes and driving innate immune response [34, 35]. Age-related increase in innate immune response may also be due to the change in heterochromatin which causes the exposure and activation of repetitive elements, including retrotransposons that has previously been observed [36]. Since we conducted bulk tissue transcriptomic analyses, we did not determine cell types that contribute to innate immune and inflammatory signaling in aging rodent skeletal muscles. Further studies aimed at identification of RNA and protein localization in tissue sections are warranted to reveal these cell types.

One might ask about mechanism: how are these genes perturbed? We found that alterations in several transcription factors could explain many of the changes observed: e.g., Runx3, Cebpa, Ikzf1, Nef2, Stat3, Esrra, and Nfyb. We did not directly query these factors in this study. We merely performed RT-qPCR amplification of selected transcription factors in all examined muscles of mice (Fig. S12) and rats (Fig. S13). These data showed that while a few transcription factors are regulated at the gene expression level, expression of others is not impacted by age (Figs. S12 and S13). Ideally, protein dynamics for these factors need to be determined. Indeed, once one does that, the next question would be how those factors might be activated, or whether the changes in transcription are epigenetically induced, making particular genes more accessible to these transcription factors. These questions will require extensive additional research and are thus left for future studies.

As to the question, “are various muscles different, similar or the same, within a species, when it comes to age-related changes?” we did see fairly similar changes across muscle types, but one could not say the changes were “the same”. For example, the diaphragm, which is constantly contracting throughout life, in order for the animal to breathe, showed the least number of age-related gene changes. This is consistent with the notion that exercise is helpful to avoid the effects of age. The changes which were seen in the diaphragm were upregulation of several inflammation pathways, including cytokine signaling and “innate immunity”, again suggestive of the idea that the muscle is undergoing a loss of heterochromatin, resulting in DNA damage [36].

Having a gene expression time course from rodents is indispensable to studying aging, but is difficult to prepare due to the cost in time and resources. Previous collections of rodent gene expression time courses made tradeoffs among the coverages of time points, tissues, sexes, and species, as well as the sample sizes for each condition [29, 30, 37]. Some of these limitations are now overcome by our data set.

After conducting this unbiased study, we next went back to the data to enquire about particular genes which have been previously shown to be upregulated in settings of skeletal muscle atrophy [21–24] (Fig. 5). We divided the figure into two sections, Fig. 5A surveys three select genes that increase coincident with denervation: Chrnd, MuSK, and Ncam1. Figure 5B surveys five genes that are associated with skeletal muscle atrophy [21]: Fboxo32, more commonly known as MAFbx or Atrogin-1; Trim63, more commonly known as MuRF1; Hdac4; Mt1, also known as Metallothionein 1—the gene is called Mt1A in the rat; Mt2 also known as Metallothionein 2 the gene is called Mt2A in the rat. The data show that markers of muscle atrophy are much more pronounced in the rat than in the mouse—there we saw a statistically significant upregulation of MuRF1, Hdac4, Mt1/Mt1A, and MT2/MT2A in the rat. None of these genes showed age-related transcriptional regulation in the mouse. Interestingly, MAFbx/Atrogin1/FbxO32 was not upregulated during sarcopenia in the mouse or the rat, distinguishing sarcopenia from more acute settings of atrophy. We had similar findings when we surveyed rat sarcopenia proteomically in the past [6], thus repeating the finding that MAFbx is not upregulated during sarcopenia in the rat, even though there is a significant loss of the skeletal muscle. As for the denervation marker genes, MuSK and acetylcholine receptor delta subunit (Chrnd) and Ncam1 are all significantly upregulated in the rat but not the mouse, indicating that the rat is functionally denervated. Prior studies do show that mice too experience NMJ changes with age [44, 46, 50], but this study shows that molecularly, there are much clearer signs of functional denervation in the rat than the mouse with age.

Sarcopenia is clearly distinct from simple acute muscle atrophy. We find signs of functional denervation, loss of mitochondrial gene expression, and significant increases in inflammation in the rat model, where the onset of age-related decline in muscle mass was evident at 21 months and further progressed by 27 months. In the mouse, age-related decline in muscle mass was less dramatic compared with the rat, and there were much fewer signs of age-related gene alterations. Although, similar to rat, mouse also displayed age-related increases in the immune and inflammatory signaling and downregulation of the ECM-related genes.

After comparing mice to rats in order to find conserved differences throughout evolution, and taking into account the prior reports on geriatric mice, one is left with the strong impression that sarcopenia is primarily a disease of inflammation, with perhaps consequent inputs due to changes in the extracellular matrix and loss of mitochondrial activity, and with an additional component contributed by the motoneuron, resulting in functional denervation—all contributing to and finally resulting in weakness, frailty, and then death. It remains to be seen if any of these processes can be pharmacologically perturbed so as to preserve muscle function in old age—we and others have already shown one instance where this was found, by using a rapalog to block some of the age-related inflammatory changes seen [51, 52]—this treatment also improved aged muscle phenotypes [51, 52]. This work needs to be followed up, asking if any of the sarcopenia-associated pathways can be counter-regulated, as this was done with pharmacological inhibition of mTORC1 signaling [30, 53], so as to continue to discover a robust set of interventions that might keep the elderly strong, healthy, and active.

## Supplementary Information

**Additional file 1: Table S1.** Number of samples for individual muscles in male rats and male and female mice. Number of samples corresponds to the number of animals. E.g. 12 muscles are collected from 12 animals. **Table S2.** Fold change and adjusted *p* values of age-related genes in skeletal muscles of rats and mice. **Table S3.** Probe and primer sequences used for RT-qPCR in mice. Highlighted genes were used as reference genes. **Table S4.** Probe and primer sequences used for RT-qPCR in rats. Highlighted genes were used as reference genes. **Figure S1.** Numbers of age-related genes under a stringent cutoff. **Figure S2.** Gastrocnemius, tibialis anterior and soleus muscle weights in male and female C57Bl6J mice (A, B) and male Sprague Dawley rats (C). **Figure S3.** Numbers of age-related genes in rat muscles, using lower animal numbers. **Figure S4.** Numbers of linear and logistic age-related genes in diaphragm, gastrocnemius, soleus and tibialis anterior muscles from female mice. **Figure S5.** Under stricter examination, rat muscles still enrich for more age-related up-regulated pathways. **Figure S6.** Age-related genes in male rats and male mice that are associated with immune (A) and mitochondrial (B) pathways. **Figure S7.** Pathways enriched by age-related genes that were shared between male and female mice. **Figure S8.** Under stricter examination, rat muscles still enrich for more age-related down-regulated pathways. **Figure S9.** Transcription factors (TFs) associated with pathways enriched by age-related genes. **Figure S10.** RT-qPCR validation of top five up- and down-regulated genes in skeletal muscles from male (A and B) and female (C and D) mice. **Figure S11.** RT-qPCR validation of top five up- and down-regulated genes in skeletal muscles from rats. **Figure S12.** RT-qPCR validation of transcription factors identified in mice (selected from Figure S9A). **Figure S13.** RT-qPCR validation of transcription factors identified in rats (selected from Figure S9B).

## Data Availability

All fastqs are available at GEO under accession number GSE226117 and GSE226118.
