# Supplementary material for: Age-related gene expression signatures from limb skeletal muscles and the diaphragm in mice and rats reveal common and species-specific changes
Source: Skelet Muscle. 2023 Jul 12;13:11. doi: 10.1186/s13395-023-00321-3 (PMC10337157; doi:10.1186/s13395-023-00321-3)

Supplemental Figure 10

A)

Gastrocnemius Male Mouse Top Upregulated Genes

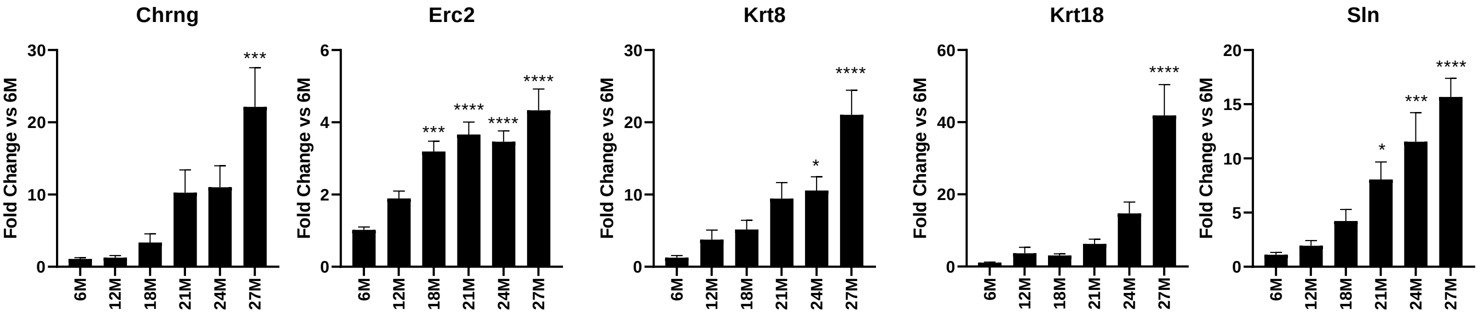

Gastrocnemius Male Mouse Top Downregulated Genes

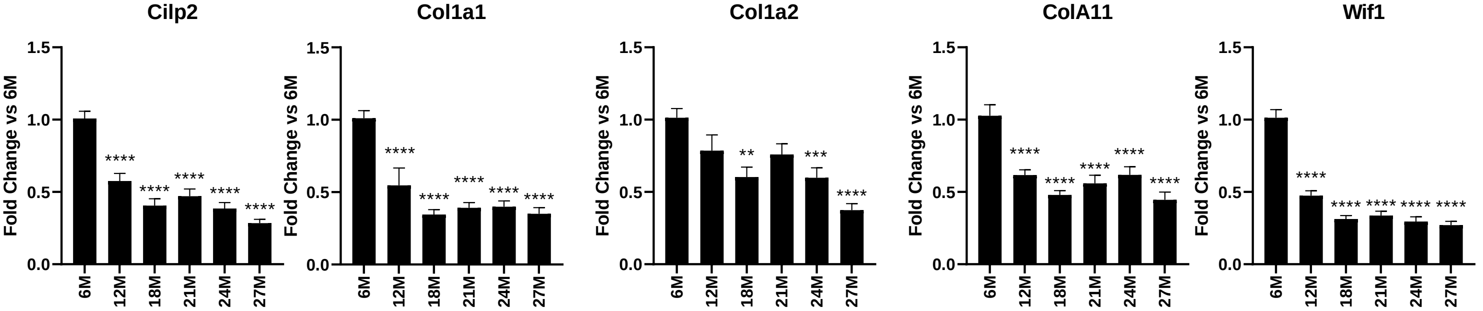

Tibialis Anterior Male Mouse Top Upregulated Genes

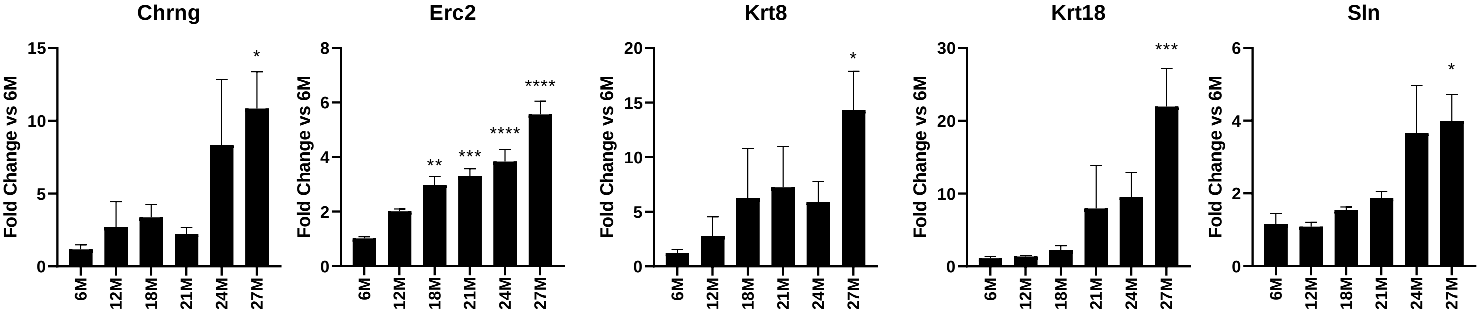

Tibialis Anterior Male Mouse Top Downregulated Genes

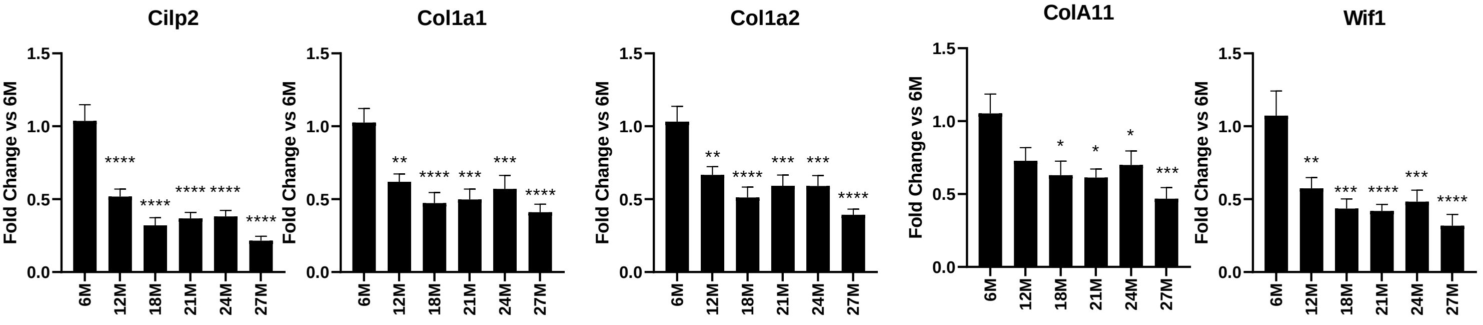

Supplemental Figure 10

B)

Soleus Male Mouse Top Upregulated Genes

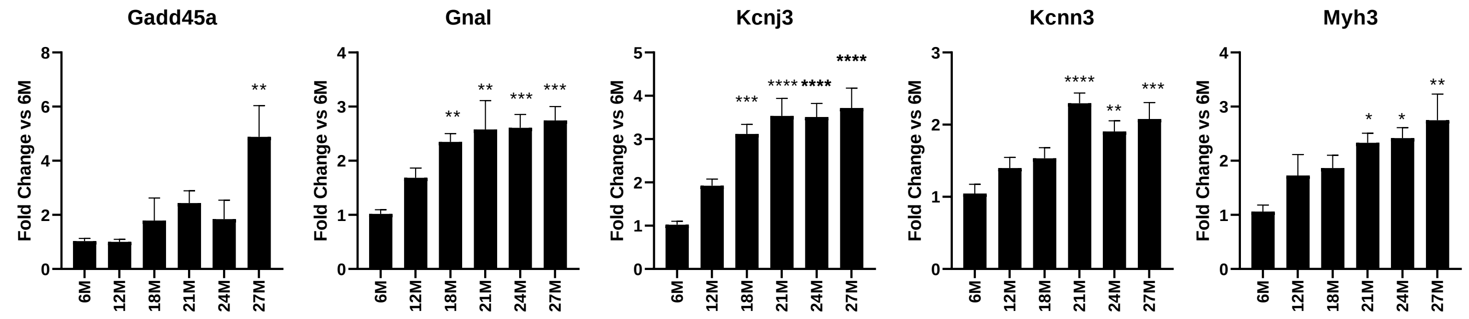

Soleus Male Mouse Top Downregulated Genes

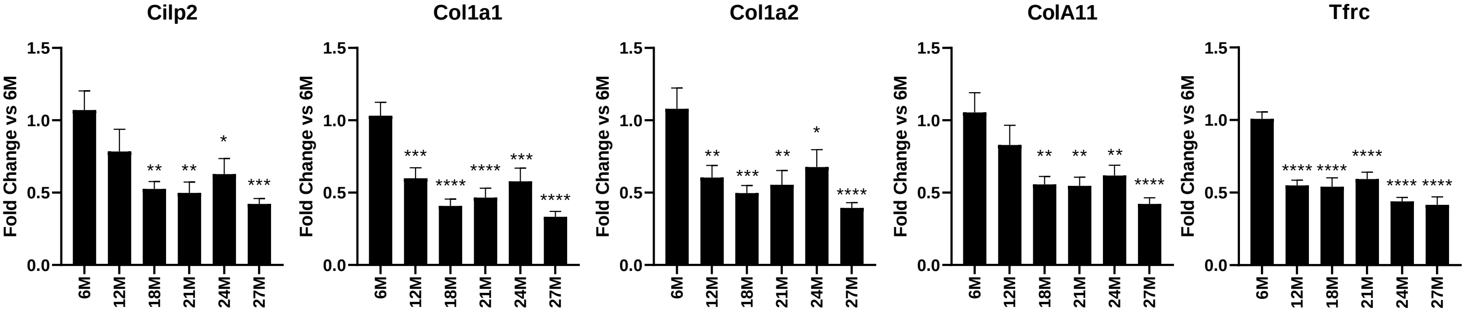

Diaphragm Male Mouse Top Upregulated Genes

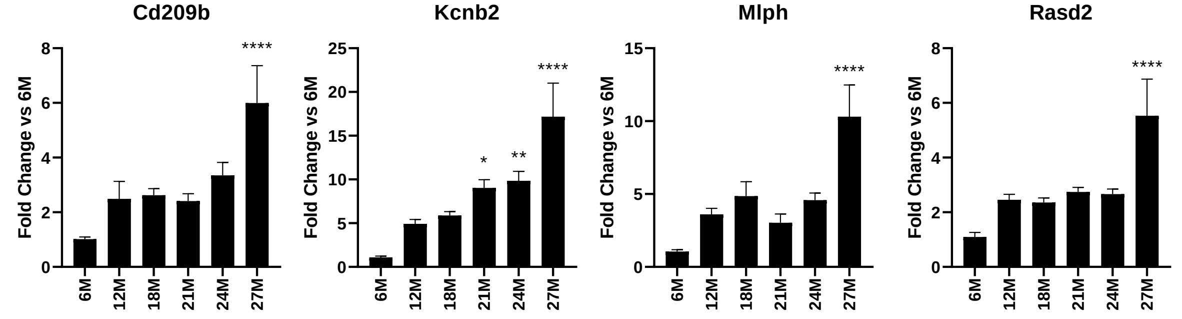

Diaphragm Male Mouse Top Downregulated Genes

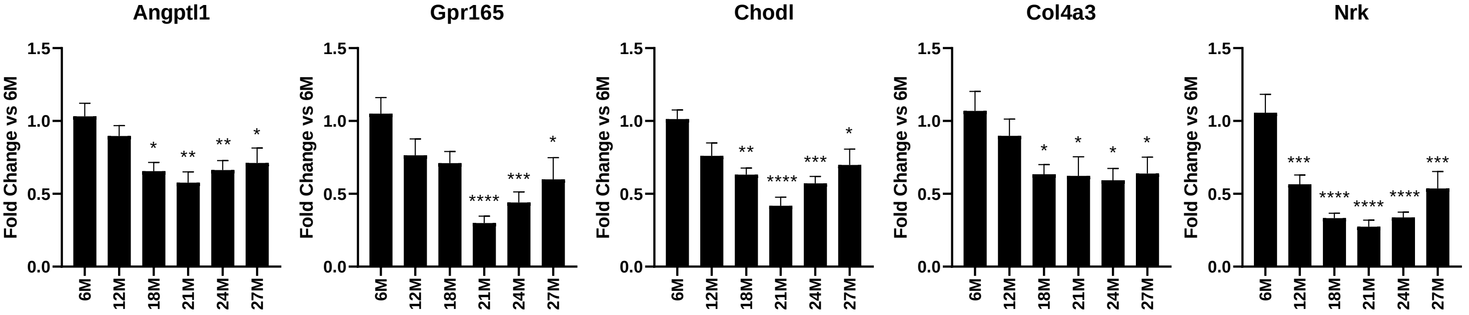

Supplemental Figure 10

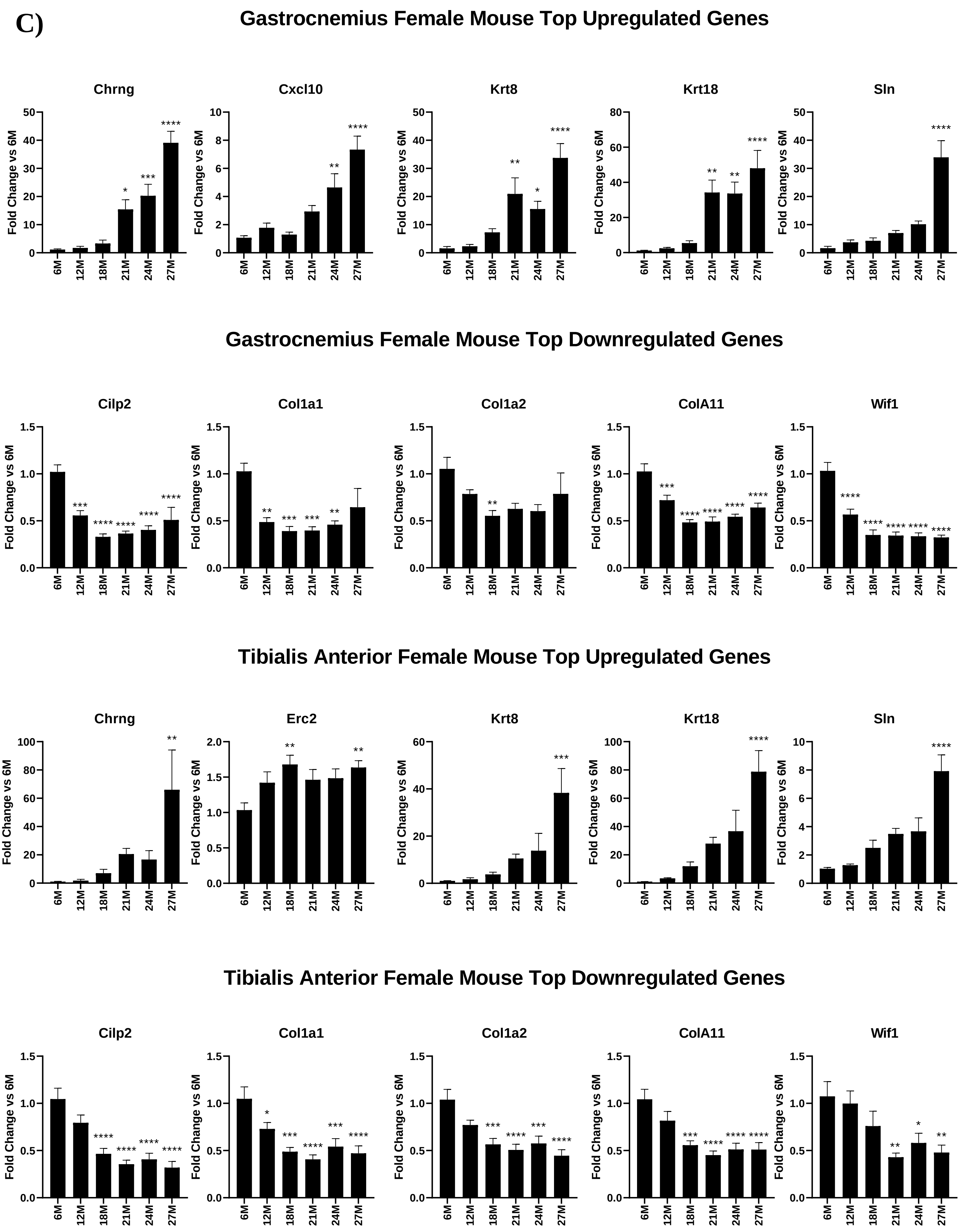

Supplemental Figure 10

D)

Soleus Female Mouse Top Upregulated Genes

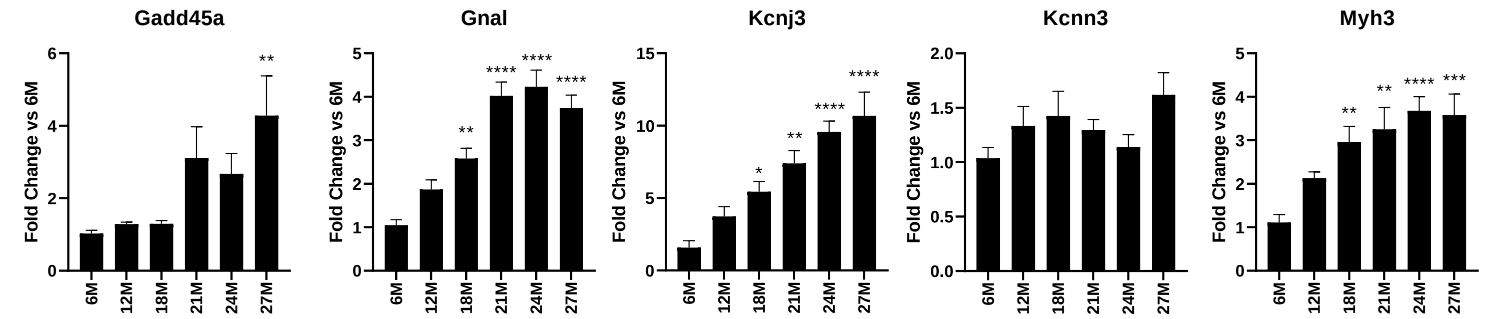

Soleus Female Mouse Top Downregulated Genes

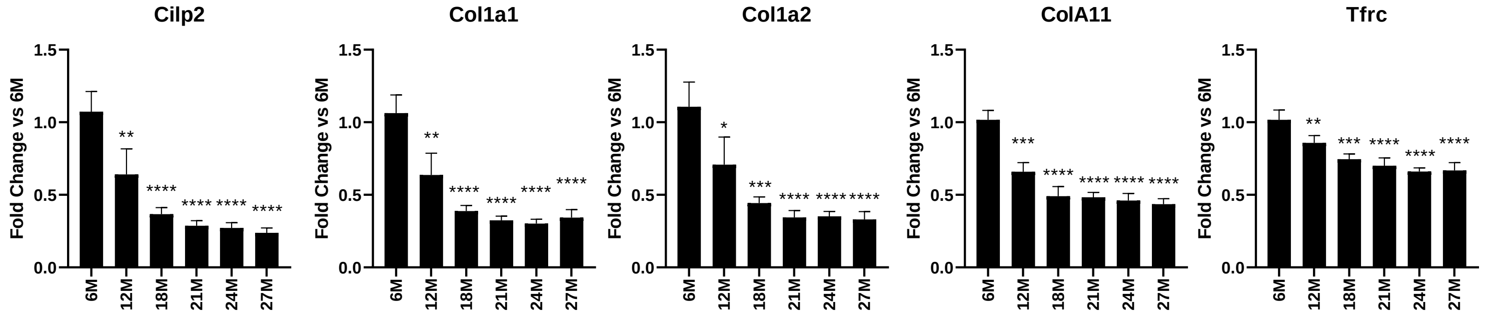

Diaphragm Female Mouse Top Upregulated Genes

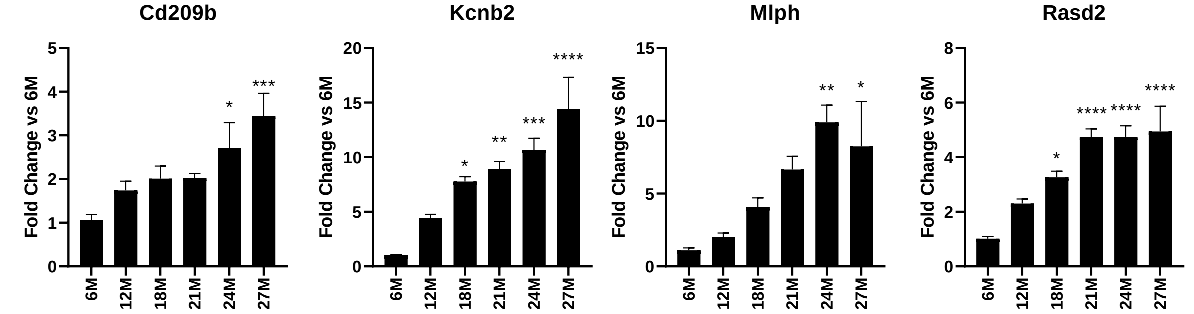

Diaphragm Female Mouse Top Downregulated Genes

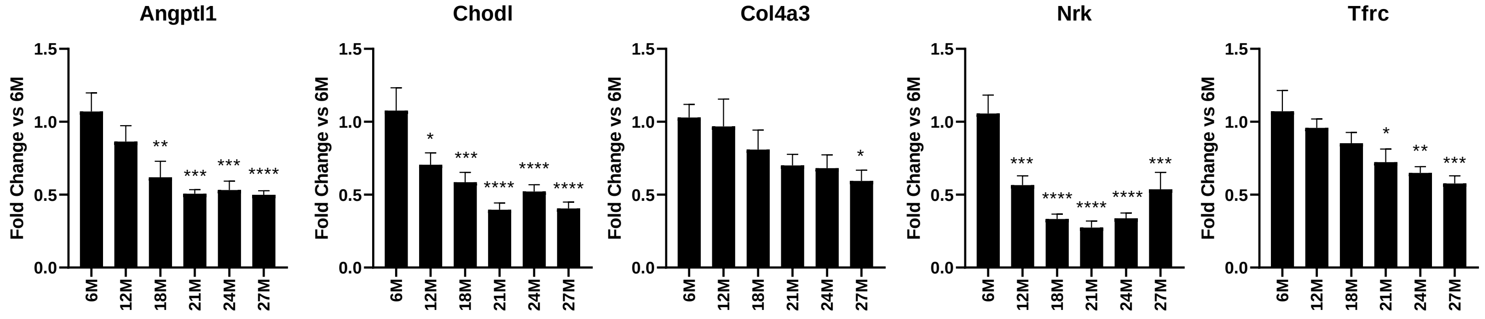

Supplemental Figure 11

A)

Gastronemius Male Rat Top Upregulated Genes

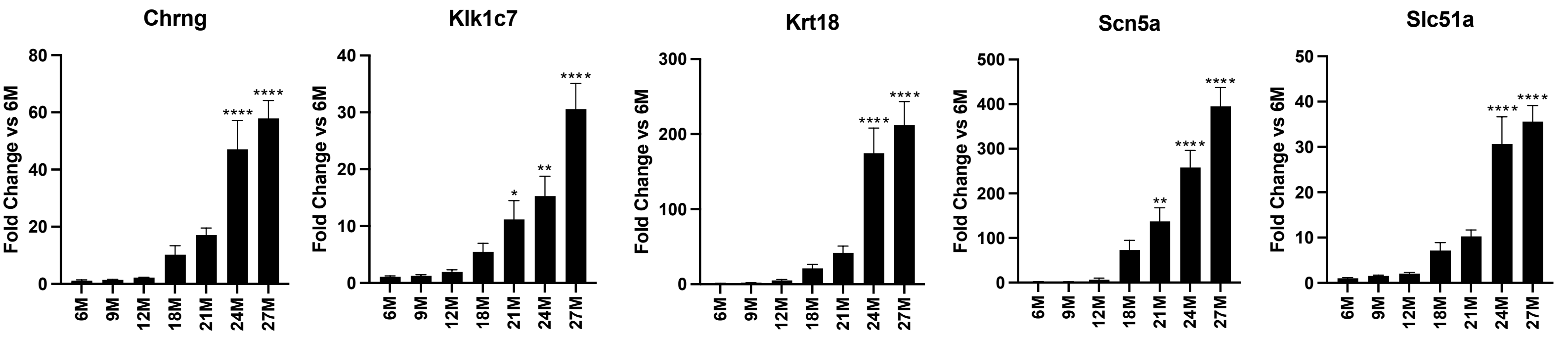

Gastronemius Male Rat Top Downregulated Genes

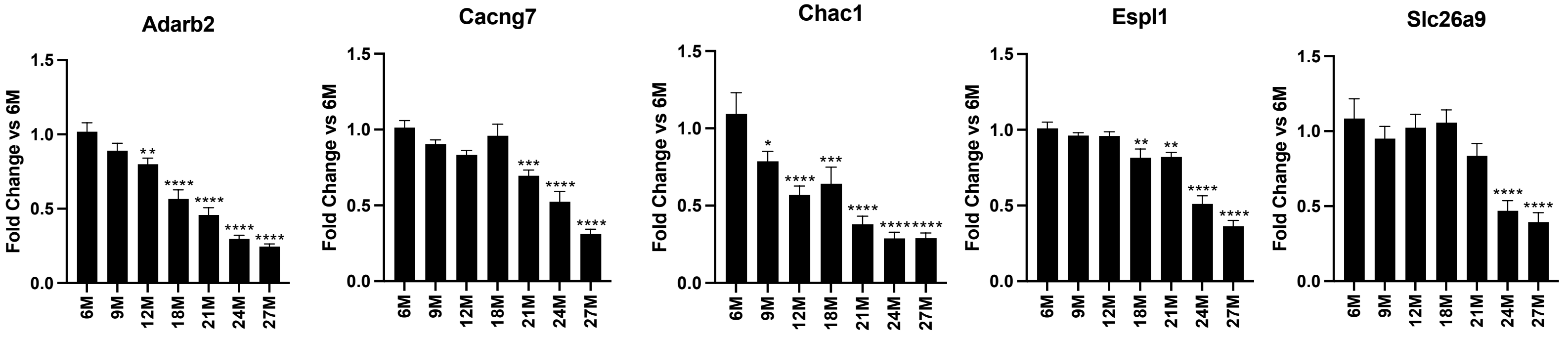

Tibialis Anterior Male Rat Top Upregulated Genes

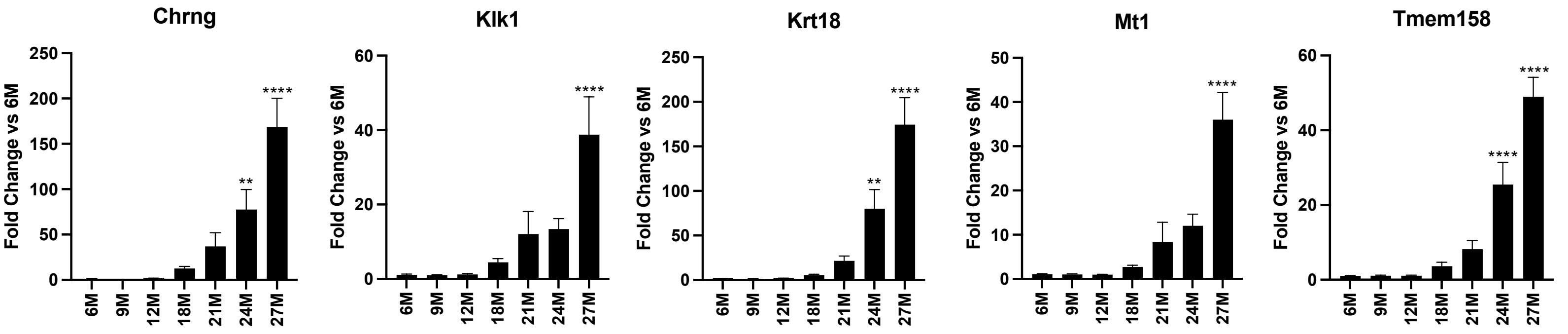

Tibialis Anterior Male Rat Top Downregulated Genes

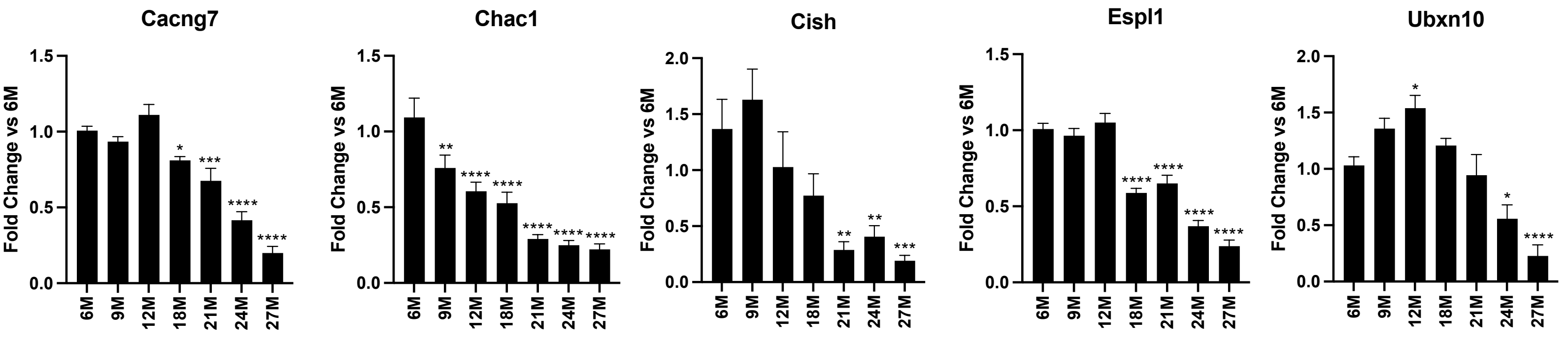

Supplemental Figure 11

B)

Soleus Male Rat Top Upregulated Genes

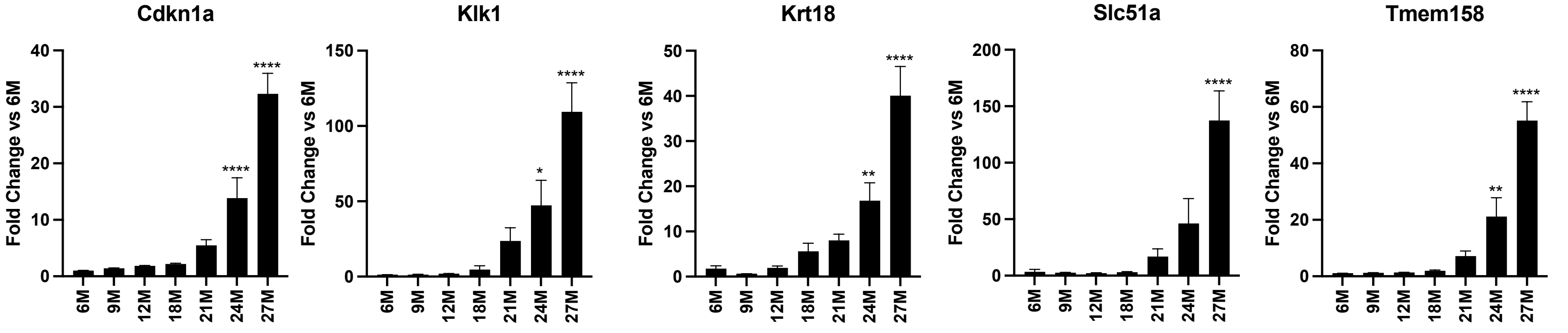

Soleus Male Rat Top Downregulated Genes

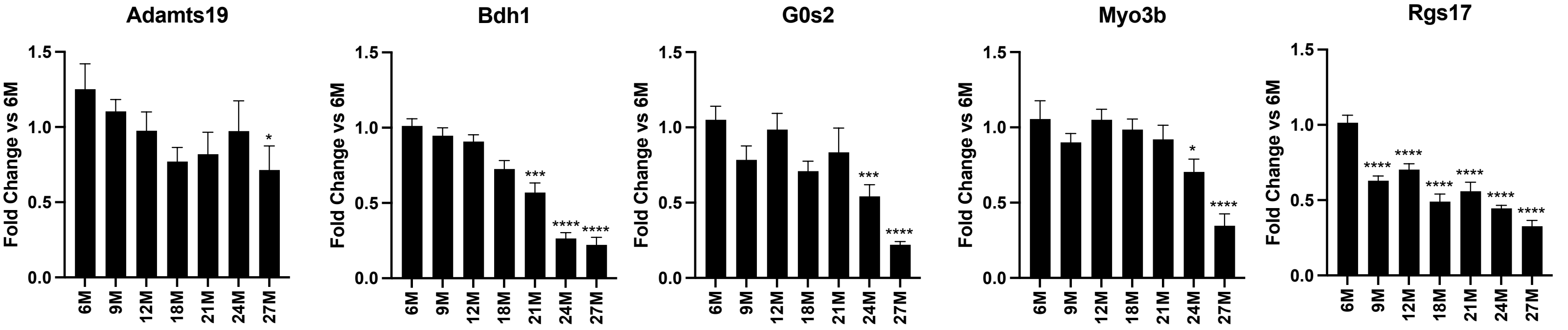

Diaphragm Male Rat Top Upregulated Genes

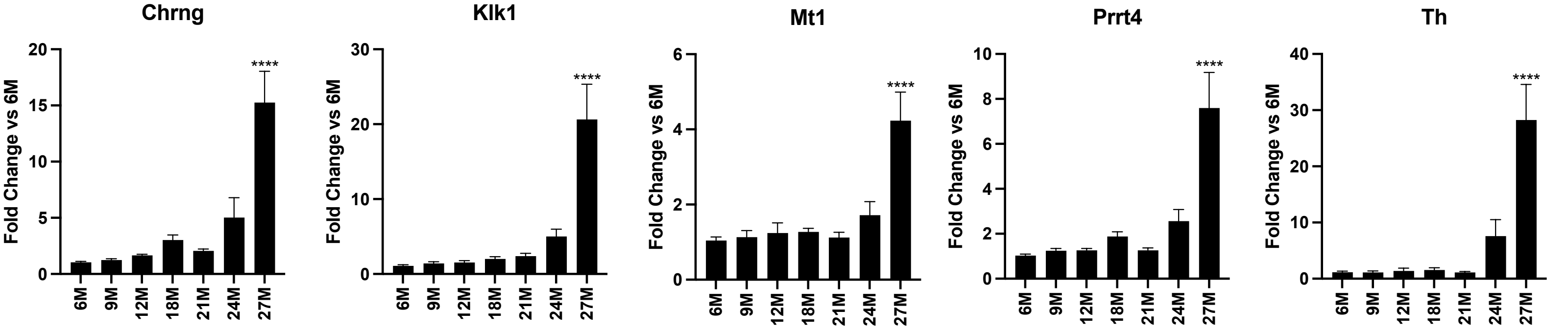

Diaphragm Male Rat Top Downregulated Genes

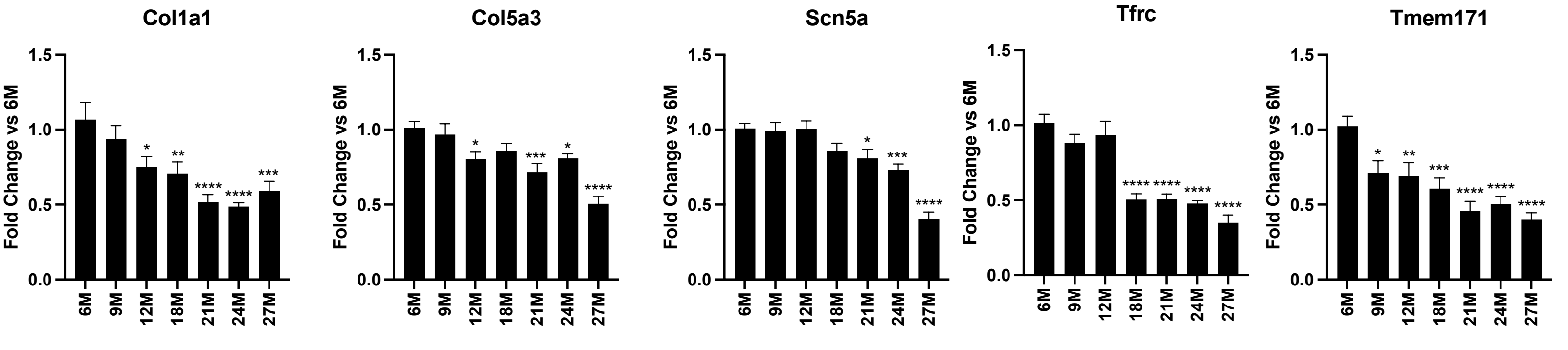

Supplemental Figure 12

A)

Gastrocnemius Male Mouse Transcription Factors

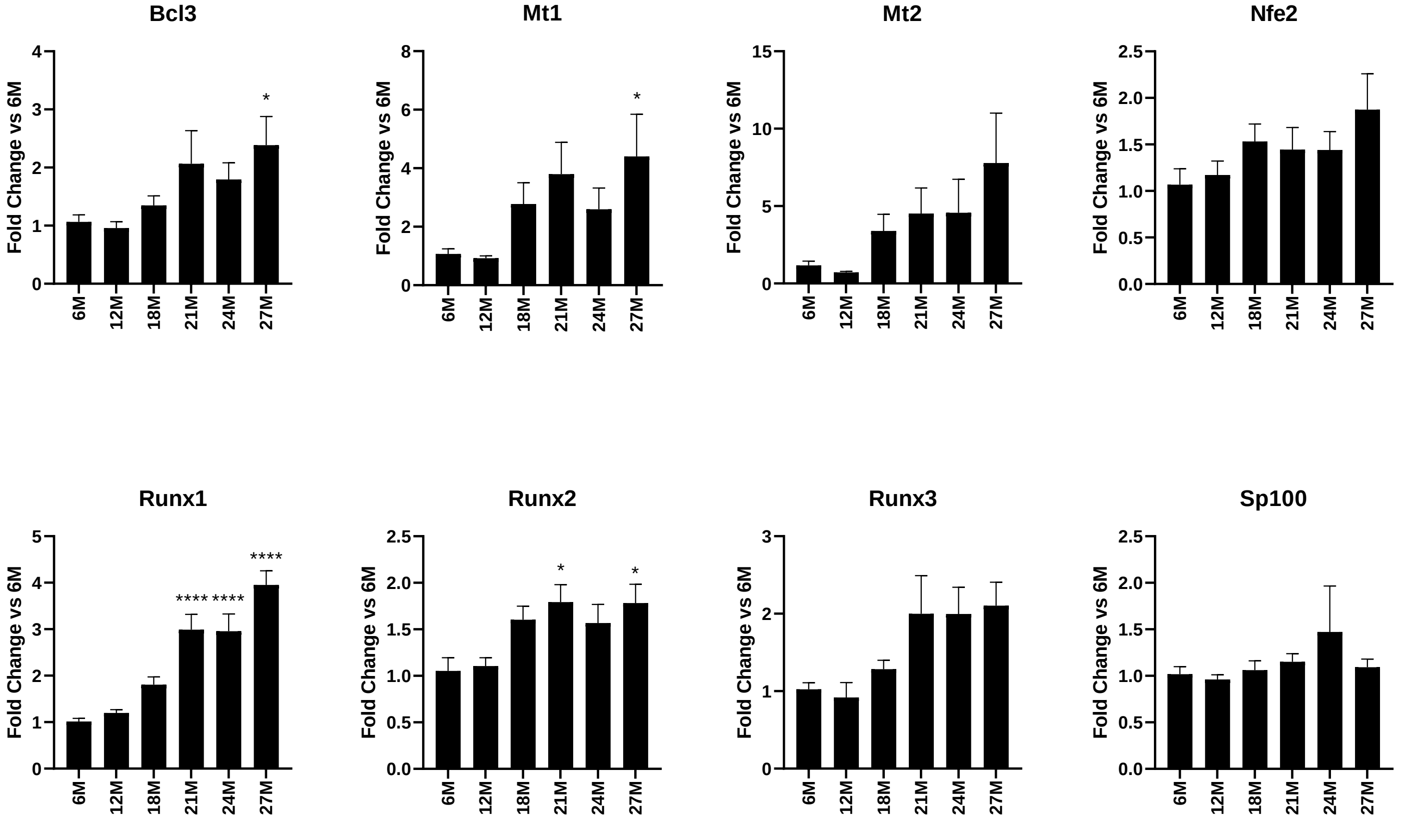

Tibialis Anterior Male Mouse Transcription Factors

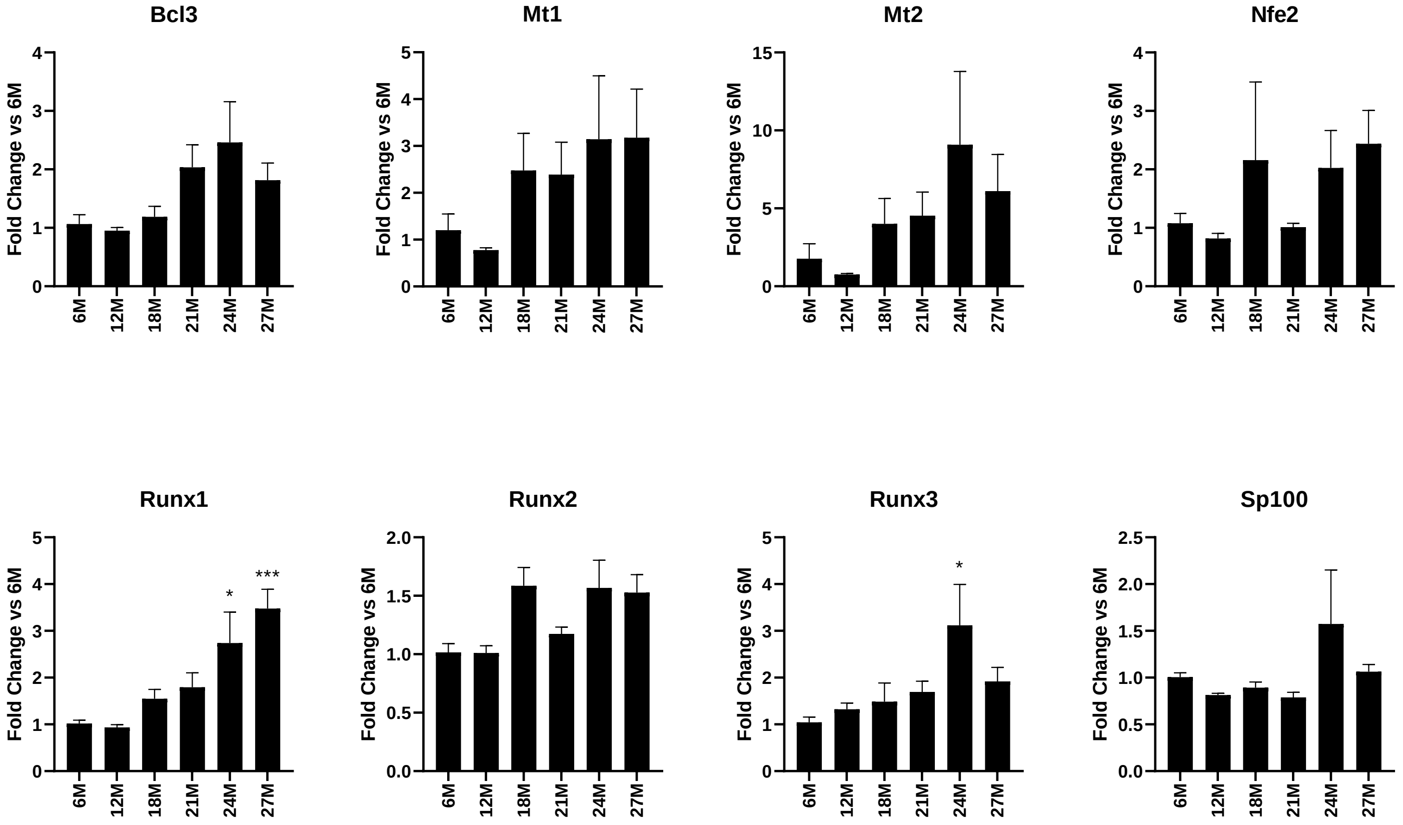

Supplemental Figure 12

B)

Soleus Male Mouse Transcription Factors

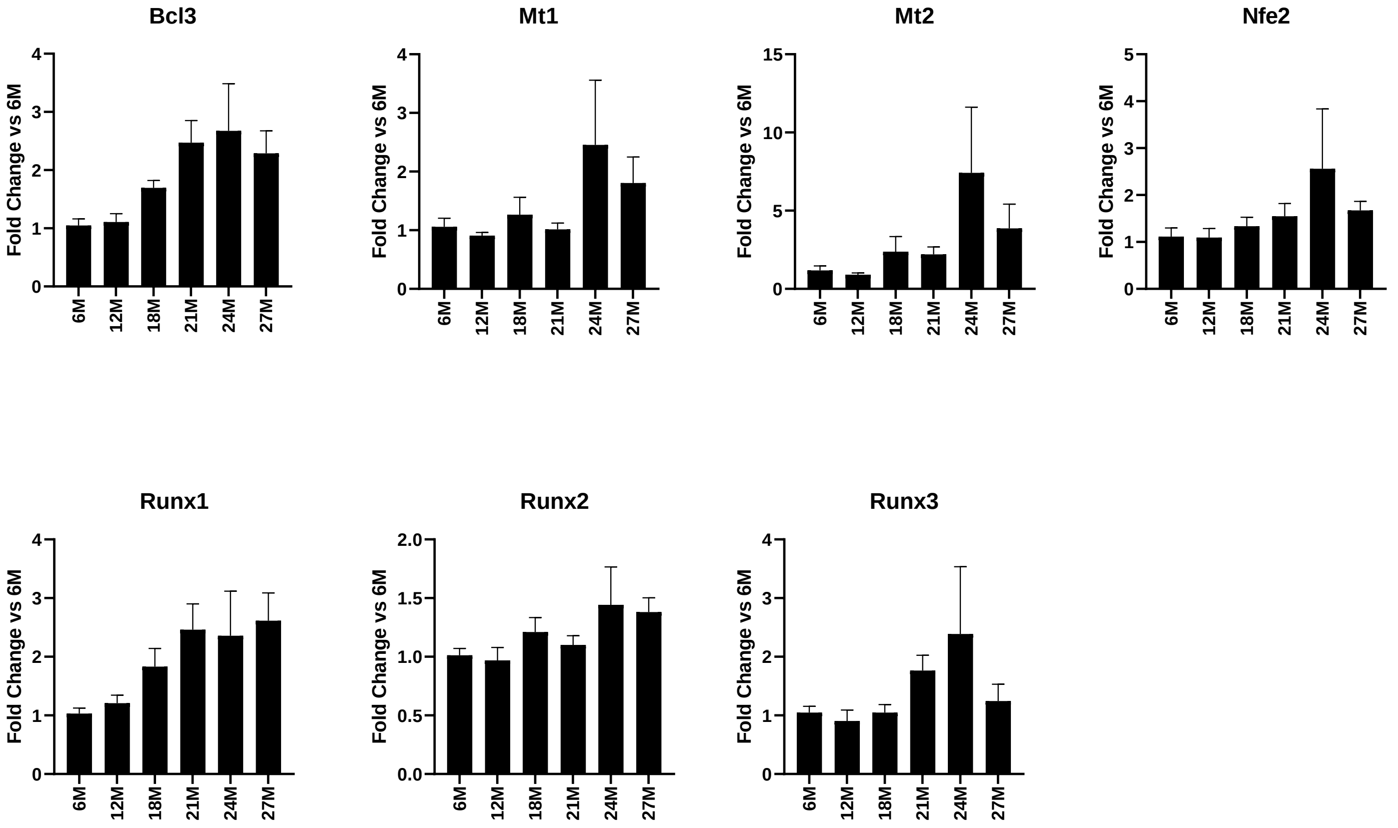

Diaphragm Male Mouse Transcription Factors

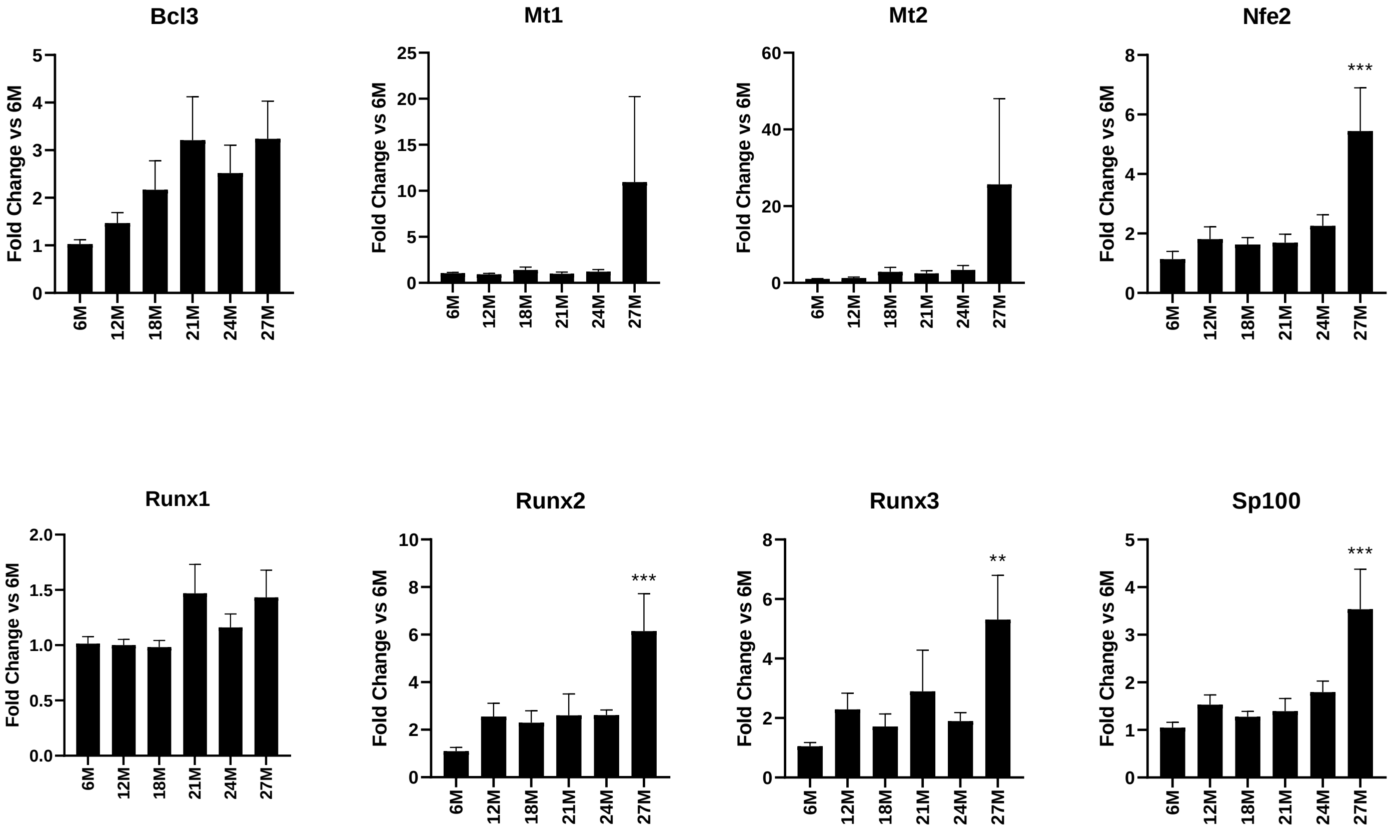

Supplemental Figure 12

C)

Gastrocnemius Female Mouse Transcription Factors

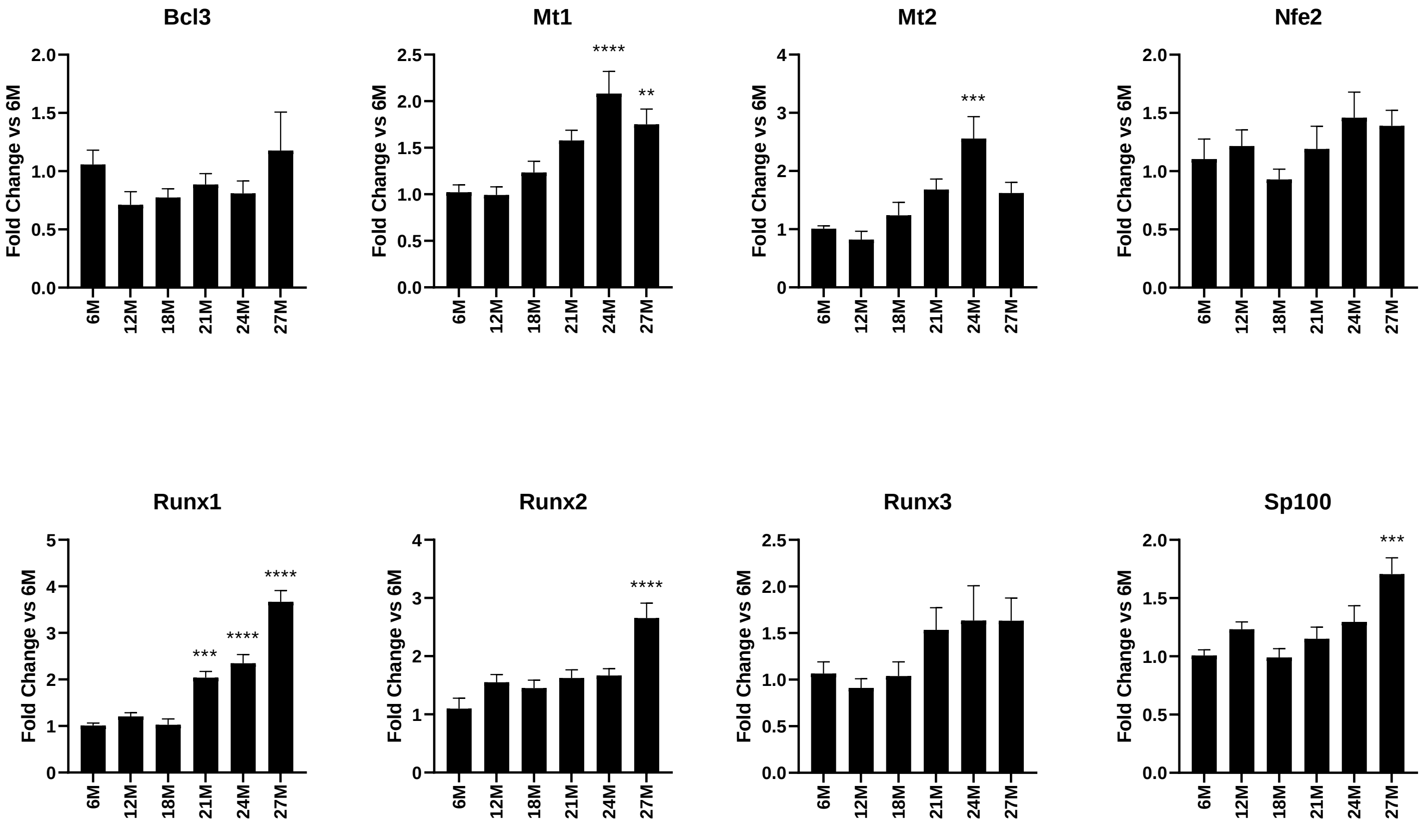

Tibialis Anterior Female Mouse Transcription Factors

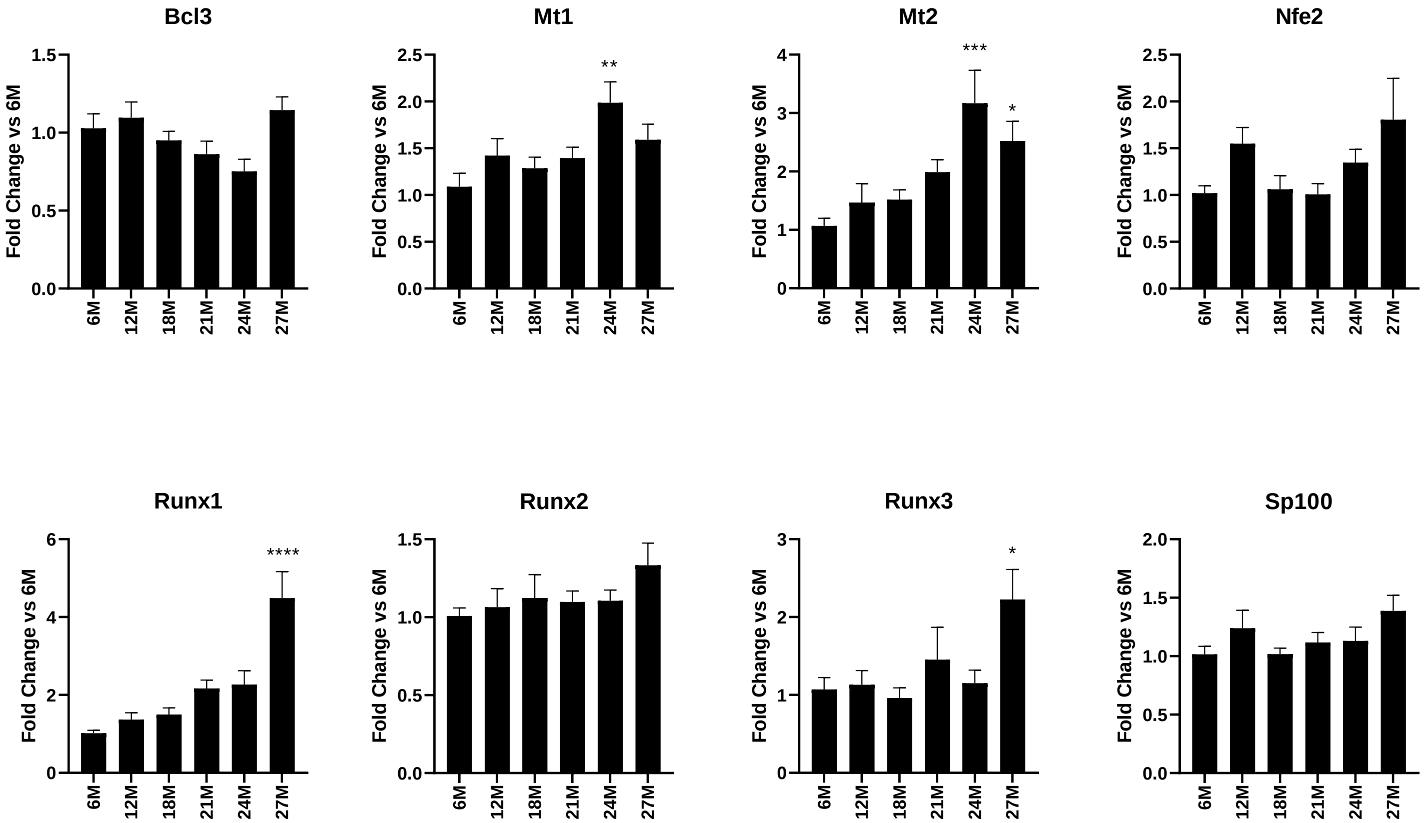

Supplemental Figure 12

D)

Soleus Female Mouse Transcription Factors

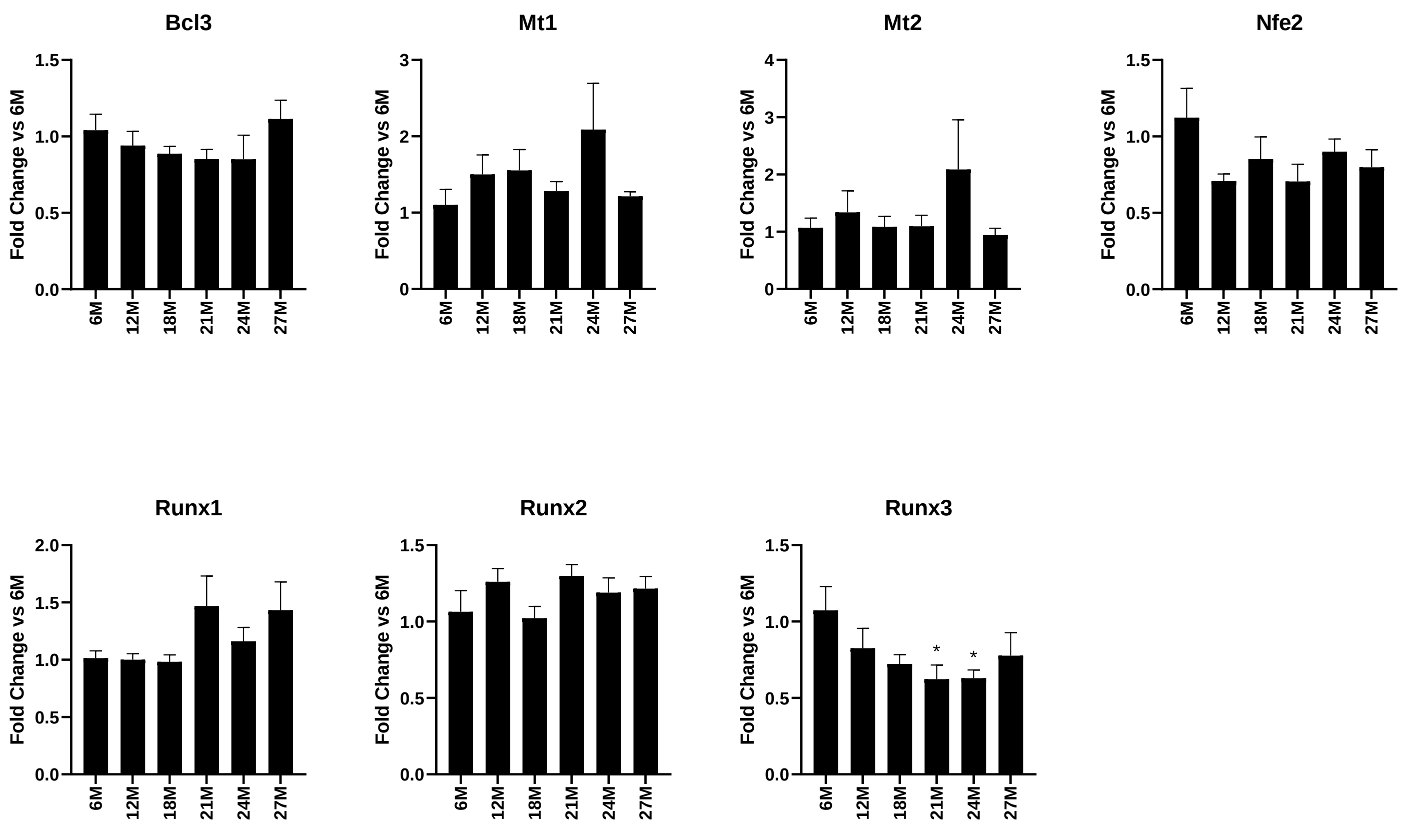

Diaphragm Female Mouse Transcription Factors

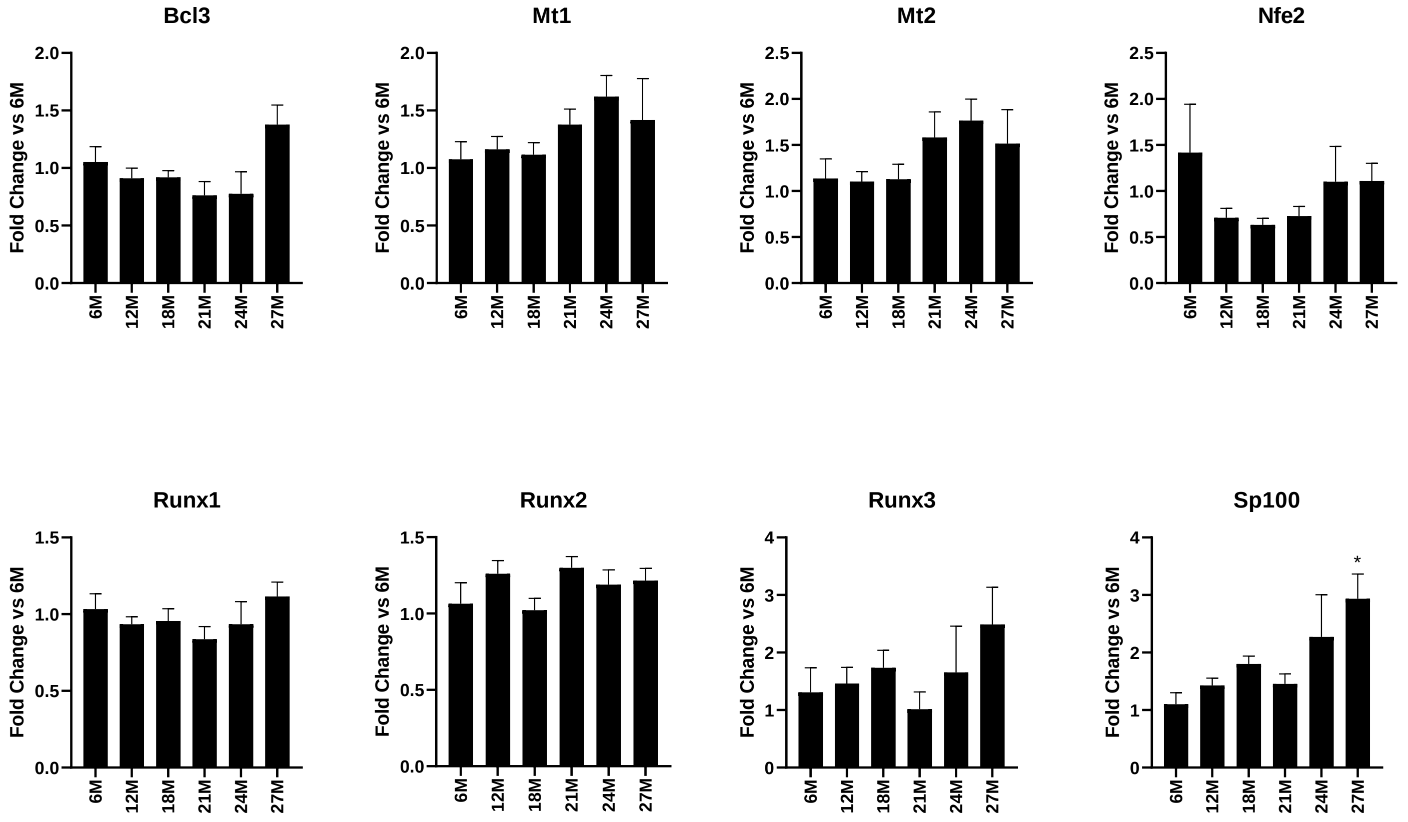

Supplemental Figure 13

A)

Gastrocnemius Male Rat Transcription Factors

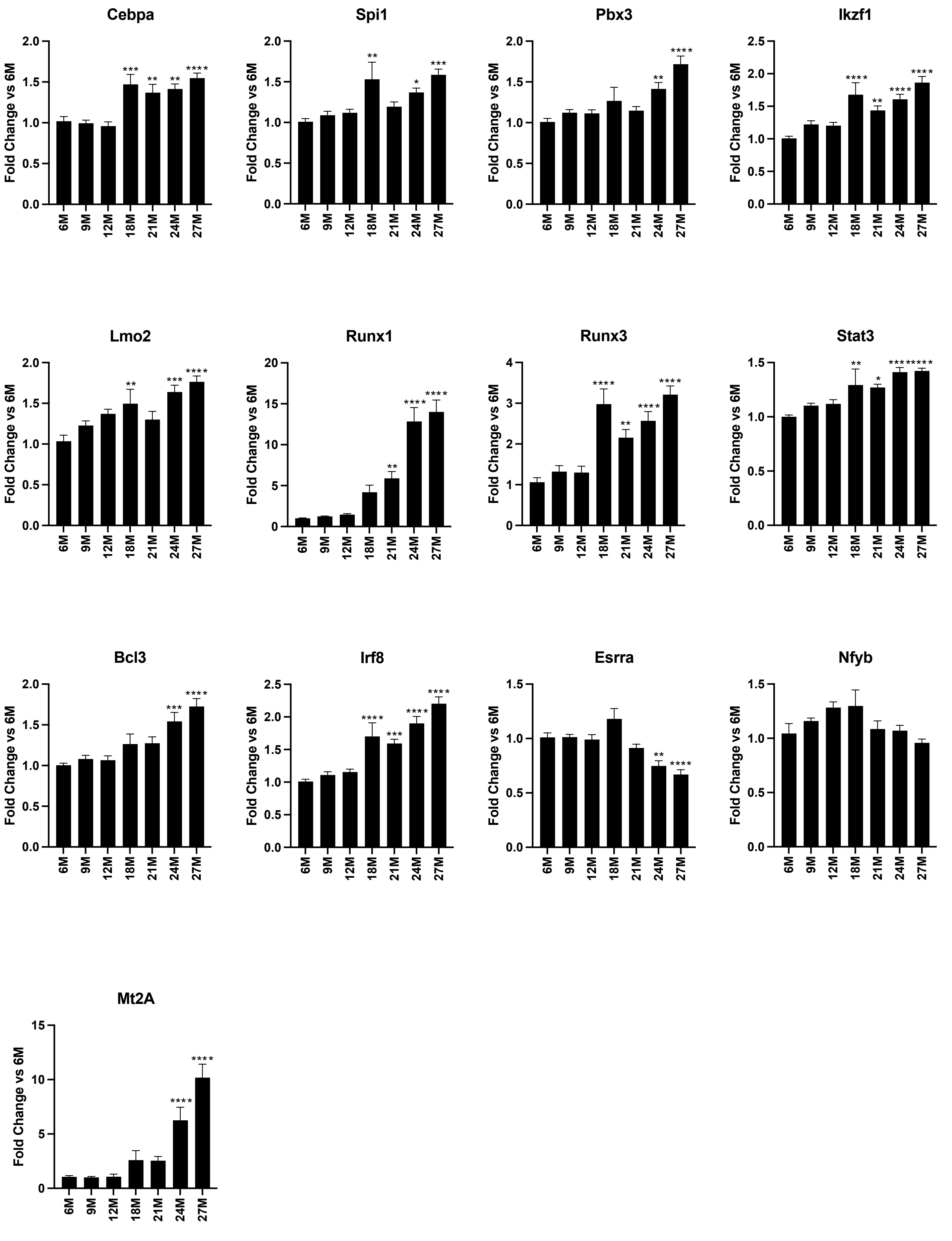

Supplemental Figure 13

B)

Tibialis Anterior Male Rat Transcription Factors

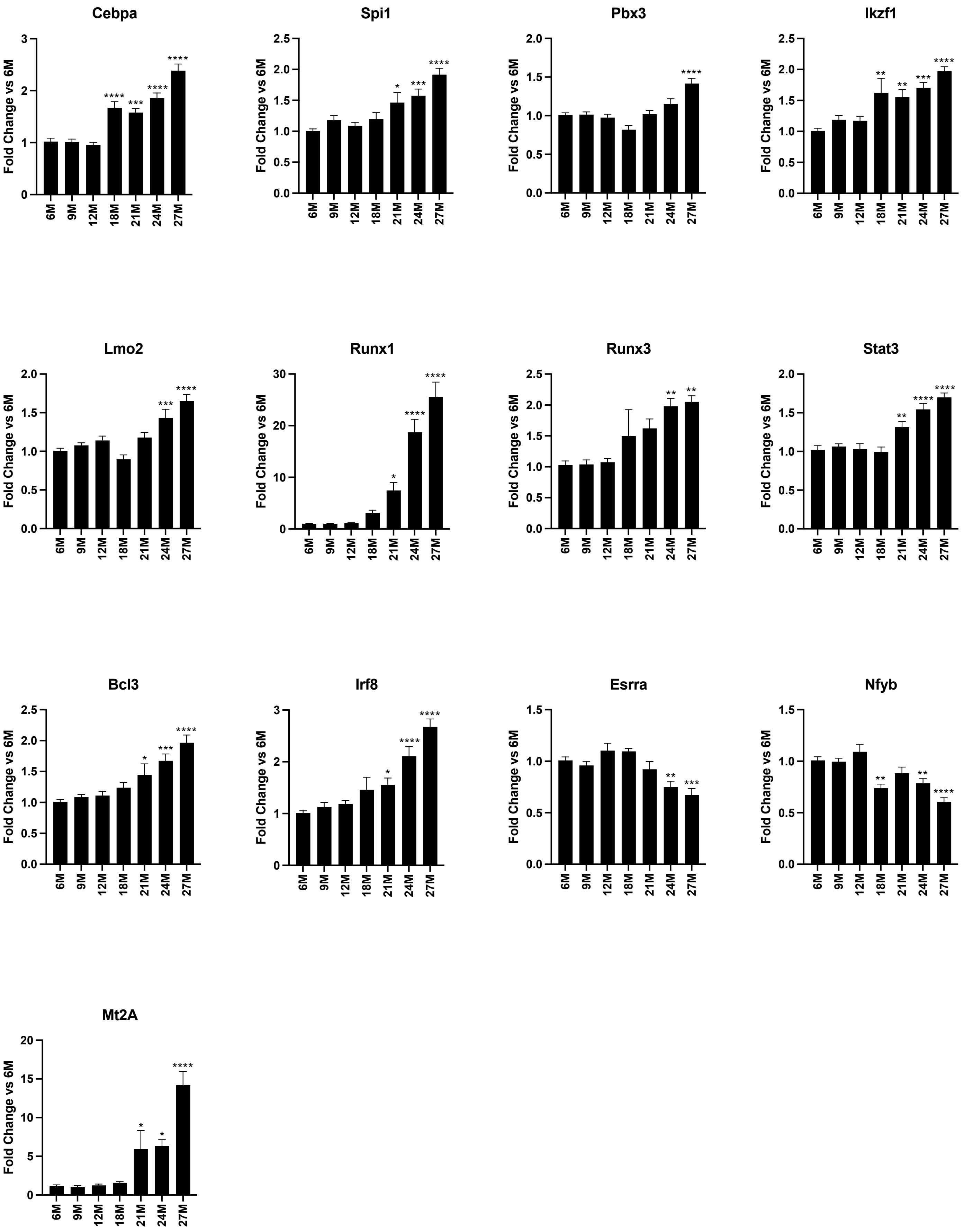

Supplemental Figure 13

C)

Soleus Male Rat Transcription Factors

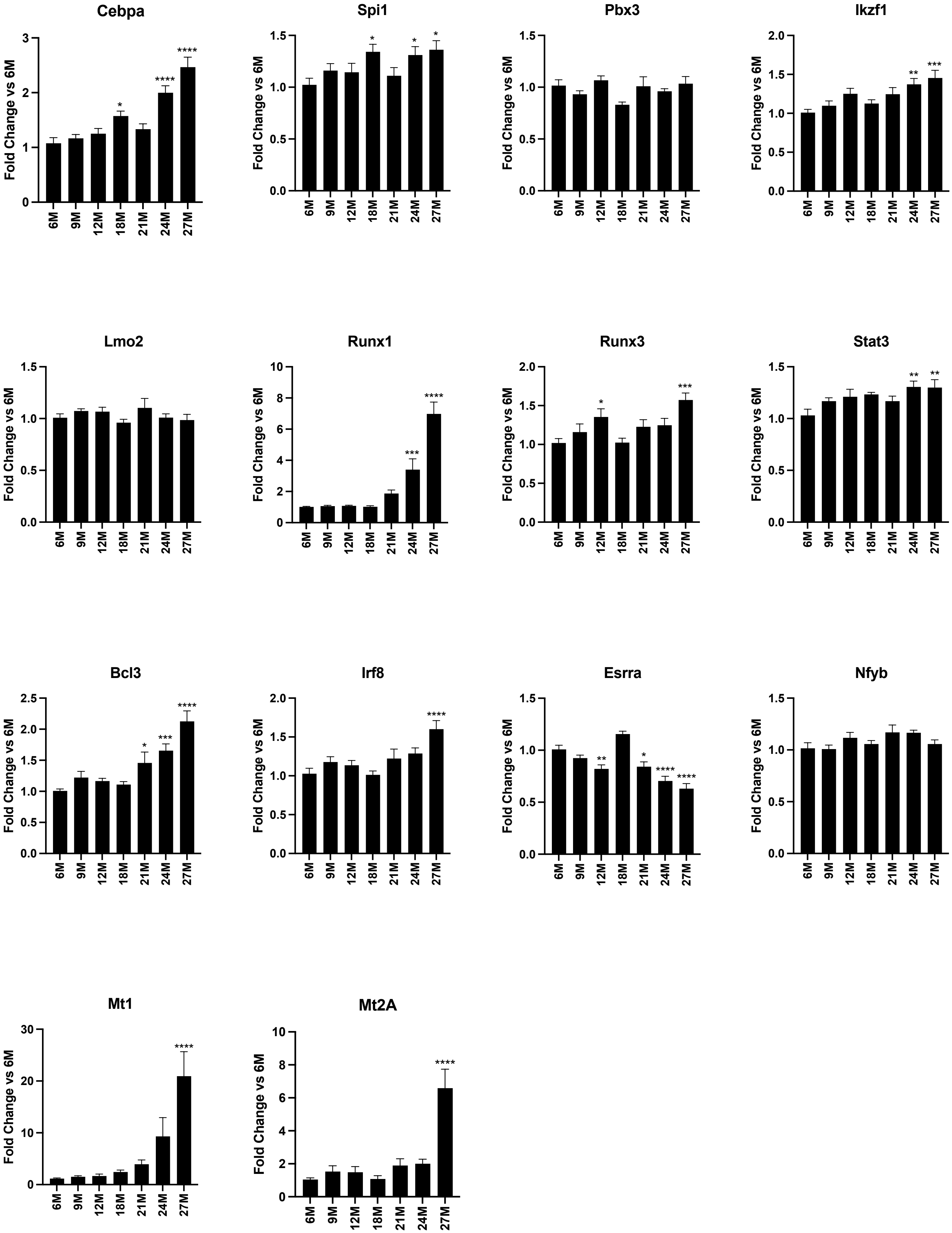

Supplemental Figure 13

D)

Diaphragm Male Rat Transcription Factors

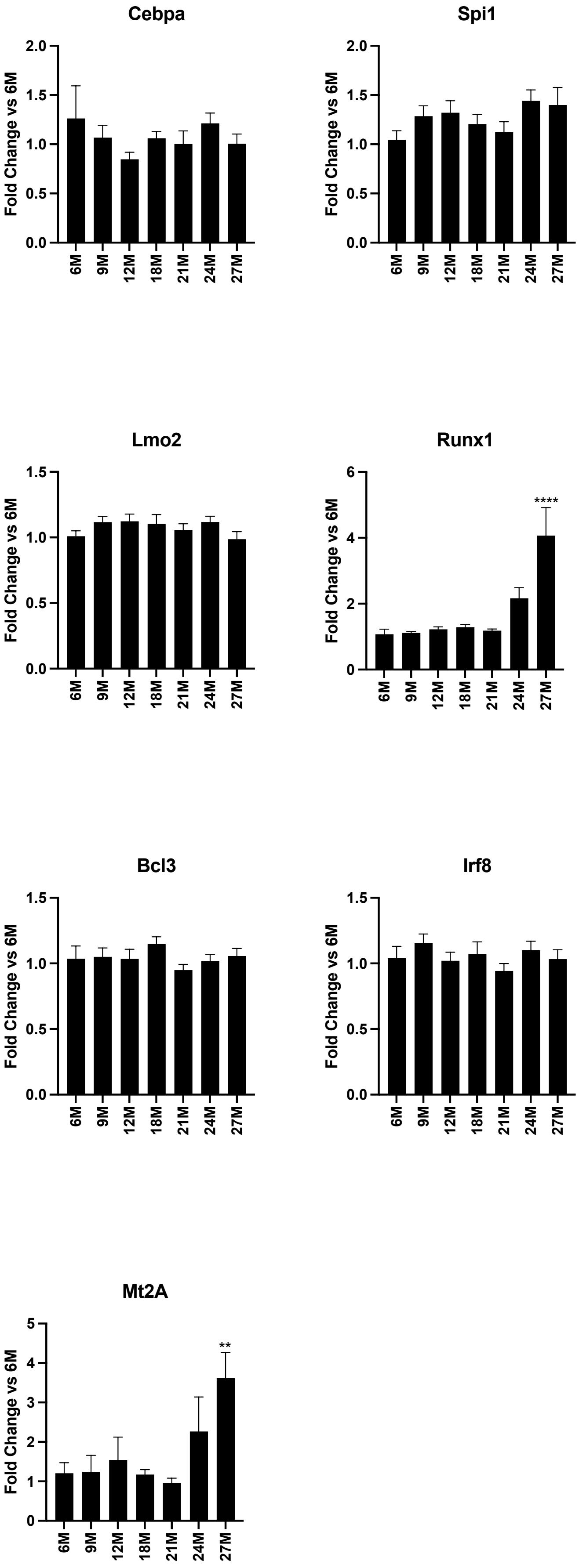

Supplement: Supplementary file 1 — Additional file 1: Table S1. Number of samples for individual muscles in male rats and male and female mice. Number of samples corresponds to the number of animals. E.g. 12 muscles are collected from 12 animals. Table S2. Fold change and adjusted p values of age-related genes in skeletal muscles of rats and mice. Table S3. Probe and primer sequences used for RT-qPCR in mice. Highlighted genes were used as reference genes. Table S4. Probe and primer sequences used for RT-qPCR in rats. Highlighted genes were used as reference genes. Figure S1. Numbers of age-related genes under a stringent cutoff. Figure S2. Gastrocnemius, tibialis anterior and soleus muscle weights in male and female C57Bl6J mice (A, B) and male Sprague Dawley rats (C). Figure S3. Numbers of age-related genes in rat muscles, using lower animal numbers. Figure S4. Numbers of linear and logistic age-related genes in diaphragm, gastrocnemius, soleus and tibialis anterior muscles from female mice. Figure S5. Under stricter examination, rat muscles still enrich for more age-related up-regulated pathways. Figure S6. Age-related genes in male rats and male mice that are associated with immune (A) and mitochondrial (B) pathways. Figure S7. Pathways enriched by age-related genes that were shared between male and female mice. Figure S8. Under stricter examination, rat muscles still enrich for more age-related down-regulated pathways. Figure S9. Transcription factors (TFs) associated with pathways enriched by age-related genes. Figure S10. RT-qPCR validation of top five up- and down-regulated genes in skeletal muscles from male (A and B) and female (C and D) mice. Figure S11. RT-qPCR validation of top five up- and down-regulated genes in skeletal muscles from rats. Figure S12. RT-qPCR validation of transcription factors identified in mice (selected from Figure S9A). Figure S13. RT-qPCR validation of transcription factors identified in rats (selected from Figure S9B). [file 13395_2023_321_MOESM1_ESM.zip › Fig S10_S11_S12_S13.pdf]
