# Supplementary material for: Age-related gene expression signatures from limb skeletal muscles and the diaphragm in mice and rats reveal common and species-specific changes
Source: Skelet Muscle. 2023 Jul 12;13:11. doi: 10.1186/s13395-023-00321-3 (PMC10337157; doi:10.1186/s13395-023-00321-3)
Supplement: Supplementary file 1 — Additional file 1: Table S1. Number of samples for individual muscles in male rats and male and female mice. Number of samples corresponds to the number of animals. E.g. 12 muscles are collected from 12 animals. Table S2. Fold change and adjusted p values of age-related genes in skeletal muscles of rats and mice. Table S3. Probe and primer sequences used for RT-qPCR in mice. Highlighted genes were used as reference genes. Table S4. Probe and primer sequences used for RT-qPCR in rats. Highlighted genes were used as reference genes. Figure S1. Numbers of age-related genes under a stringent cutoff. Figure S2. Gastrocnemius, tibialis anterior and soleus muscle weights in male and female C57Bl6J mice (A, B) and male Sprague Dawley rats (C). Figure S3. Numbers of age-related genes in rat muscles, using lower animal numbers. Figure S4. Numbers of linear and logistic age-related genes in diaphragm, gastrocnemius, soleus and tibialis anterior muscles from female mice. Figure S5. Under stricter examination, rat muscles still enrich for more age-related up-regulated pathways. Figure S6. Age-related genes in male rats and male mice that are associated with immune (A) and mitochondrial (B) pathways. Figure S7. Pathways enriched by age-related genes that were shared between male and female mice. Figure S8. Under stricter examination, rat muscles still enrich for more age-related down-regulated pathways. Figure S9. Transcription factors (TFs) associated with pathways enriched by age-related genes. Figure S10. RT-qPCR validation of top five up- and down-regulated genes in skeletal muscles from male (A and B) and female (C and D) mice. Figure S11. RT-qPCR validation of top five up- and down-regulated genes in skeletal muscles from rats. Figure S12. RT-qPCR validation of transcription factors identified in mice (selected from Figure S9A). Figure S13. RT-qPCR validation of transcription factors identified in rats (selected from Figure S9B). [file 13395_2023_321_MOESM1_ESM.zip › Supplementary Tables 1 and Supplementary Figure Legends.docx]

**Supplemental table1 and Supplementary figure legends**

**Table S1.** Number of samples for individual muscles in male rats and male and female mice. Number of samples corresponds to the number of animals. E.g. 12 muscles are collected from 12 animals.

**Number of samples**

|  | 6 months | 9 months | 12 months | 18 months | 21 months | 24 months | 27 months |  |
| --- | --- | --- | --- | --- | --- | --- | --- | --- |
| Rat diaphragm, male | 12 | 11 | 12 | 12 | 12 | 12 | 12 |  |
| Rat gastrocnemius, male | 10 | 12 | 11 | 11 | 9 | 9 | 11 |  |
| Rat soleus, male | 12 | 12 | 12 | 12 | 12 | 12 | 12 |  |
| Rat tibialis anterior, male | 12 | 12 | 12 | 12 | 13 | 12 | 12 |  |
| Mouse diaphragm, male | 8 | 0 | 7 | 6 | 6 | 10 | 10 |  |
| Mouse gastrocnemius, male | 8 | 0 | 8 | 8 | 8 | 10 | 10 |  |
| Mouse soleus, male | 8 | 0 | 8 | 8 | 8 | 10 | 10 |  |
| Mouse tibialis anterior, male | 8 | 0 | 8 | 8 | 8 | 10 | 10 |  |
| Mouse diaphragm, female | 8 | 0 | 7 | 7 | 8 | 10 | 10 |  |
| Mouse gastrocnemius, female | 8 | 0 | 8 | 8 | 8 | 10 | 10 |  |
| Mouse soleus, female | 8 | 0 | 8 | 8 | 8 | 9 | 10 |  |
| Mouse tibialis anterior, female | 8 | 0 | 8 | 7 | 8 | 10 | 10 |  |

**Table S2.** Fold change and adjusted p values of age-related genes in skeletal muscles of rats and mice*.*

**Table S3.** Probe and primer sequences used for RT-qPCR in mice. Highlighted genes were used as reference genes.

**Table S4.** Probe and primer sequences used for RT-qPCR in rats. Highlighted genes were used as reference genes.

**Figure S1. Numbers of age-related genes under a stringent cutoff.** Age-related genes identified in the main manuscript (**Figure 2B**) were derived using a “default cutoff”, whereby we required the BIC score of age-related genes to be smaller than the BIC score of the null model by more than 6. In addition, we required that the BIC score of logistic genes was smaller than that of linear genes by more than 2. Here we employed a more “stringent cutoff”, where we increased the two aforementioned numbers to 10 and 4 respectively.

**Figure S2. Gastrocnemius, tibialis anterior and soleus muscle weights in male and female C57Bl6J mice (A,B) and male Sprague Dawley rats (C).** The X axes show the age of animals (months) and the Y axes show muscle weights (grams). For mice, each value represents the average of two muscle weights from the left and the right leg. For rats, each value represents the absolute muscles weight from the left leg. Data are mean ± standard deviation. Statistical significance was determined by a one-way ANOVA followed by Tukey’s multiple comparison tests. The black lines over the data bars show comparisons between 24 month and all other ages. In addition, the red lines in C show pairwise comparisons between two closest ages (e.g. 6 to 9; 9 to 12; 12 to 18 etc.) using unpaired t-tests. Asterisks denote significance as follows: * p<0.5; ** p<0.01; ** p<0.001; *** p<0.0001

**Figure S3. Numbers of age-related genes in rat muscles, using lower animal numbers.** For each rat skeletal muscle, we randomly picked 8 animals from each age group and searched for age-related genes. This strategy was used to match animal numbers used in rats with those in mice. After down sizing the groups, each muscle carries 2,016 age-related genes on average.

**Figure S4. Numbers of linear and logistic age-related genes in diaphragm, gastrocnemius, soleus and tibialis anterior muscles from female mice. (A)** Numbers of age-related genes in each skeletal muscle. Red boxes represent up-regulated genes and green boxes represent down-regulated genes**. (B)** Venn diagrams showing overlaps (shared genes) between age-related genes in male and female muscles. P values are based on Fisher’s exact test and show that the overlap is significant. Venn diagrams in the top panel represent up-regulated genes, and in the bottom panel, down-regulated genes. Blue circles represent males and pink circles, females.

**Figure S5. Under stricter examination, rat muscles still enrich for more age-related up-regulated pathways.** We repeated the analysis in Figure 3, relaxing the criterion for age-related genes and removing potential confounding factors in gene and/or pathway annotations between the two species. We relaxed the criterion for the age-related gene discovery by reducing the minimal fold change requirement from 1.5 to 1.25 when comparing 6 month to any older age. To make a common annotation of genes between rat and mouse, we identified ~14k genes that have the same names in rat and mouse. Age-related genes that are not present in these 14k genes were excluded. To make a common pathway set, we took the mouse pathways and removed genes that were absent in the 14k genes. Following the same approach, we could have also used the rat pathways to generate a common pathway set. However, we did not do this because either choice yields unbiased results.

**Figure S6. Age-related genes in male rats and male mice that are associated with immune (A) and mitochondrial (B) pathways. (A)** A subset of linear-up genes (30 of 259 shared by ≥ 2 tissues) enriched to “immune system” theme shown in Figure 3. Each column for each muscle corresponds to the animals age increasing from left to right. Each row is a gene and shows the log2 fold change of the average expression between animals of the given age compared with 6 months. Genes that do not satisfy criteria for “linear-up” genes in a given tissue are marked as gray. **(B)** Late-down genes (30 of 30 shared by ≥ 2 tissues) enriched to the “mitochondrial function” theme shown in Figure 4. Each column for each muscle corresponds the animal age increasing from left to right.

**Figure S7. Pathways enriched by age-related genes that were shared between male and female mice.** Similar to Figure 3, we identified pathways enriched by each class of age-related genes. We analyzed 6, 12, 18, 21, 24 and 27 month old male and female mice. Pathways enriched by linear-up, late-up, linear-down and early-down genes are depicted graphically as circles. Each column of circles, for each muscle, corresponds to the comparison between 6 month and the older age (12, 18, 21, 24 and 27 months for mice and 9, 12, 18, 21, 24 and 27 months for rats). The circle size represents the number of age-related genes enriched to each pathway: small circles denote enrichment of the pathway by 0-5 genes; medium circles, enrichment by 6-15 genes and large circles, enrichment by >15 genes. The circle color key indicates the average fold-change in the expression levels of these genes versus 6 months. Dark squares on the right-hand side of the graphical heatmap indicate that enrichment for a specific pathway was statistically significant.

**Figure S8. Under stricter examination, rat muscles still enrich for more age-related down-regulated pathways.** We repeat the analysis in Figure 4 using a lenient set of age-related genes and removing confounding factors in gene and pathway annotations. We relaxed the criterion for the age-related gene discovery by reducing the minimal fold change requirement from 1.5 to 1.25 when comparing 6 month to any older age. To make a common annotation of genes between rat and mouse, we identified ~14k genes that have the same names in rat and mouse. Age-related genes that are not present in these 14k genes were excluded. To make a common pathway set, we took the mouse pathways and removed genes that were absent in the 14k genes.

**Figure S9**. **Transcription factors (TFs) associated with pathways enriched by age-related genes. (A)** TFs associated with age-related pathways enriched in two or more rat skeletal muscles. **(B)** TFs associated with pathways shared by any skeletal muscle from male and female mice. Color represents the average Spearman correlation in expression between the TF and age-related genes that enrich to the pathway. Gray indicates that either the correlation is not significant or that none of the genes in the pathway is a known target of the given TF.

**Figure S10. RT-qPCR validation of top five up- and down-regulated genes in skeletal muscles from male (A and B) and female (C and D) mice.** Data are mean ± SEM (standard error of the mean). Data were analyzed using a one-way ANOVA followed by Dunnett’s multiple comparison tests. *p ≤ .05; **p ≤ .01; ***p ≤ .001; ****p ≤ .0001 versus 6 months, respectively.

**Figure S11. RT-qPCR validation of top five up- and down-regulated genes in skeletal muscles from rats.** Data are mean ± SEM (standard error of the mean). Data were analyzed using a one-way ANOVA followed by Dunnett’s multiple comparison tests. *p ≤ .05; **p ≤ .01; ***p ≤ .001; ****p ≤ .0001 versus 6 months, respectively.

**Figure S12. RT-qPCR validation of transcription factors identified in mice (selected from Figure S9A).** Data are mean ± SEM (standard error of the mean). Data were analyzed using a one-way ANOVA followed by Dunnett’s multiple comparison tests. *p ≤ .05; **p ≤ .01; ***p ≤ .001; ****p ≤ .0001 versus 6 months, respectively.

**Figure S13. RT-qPCR validation of transcription factors identified in rats (selected from Figure S9B).** Data are mean ± SEM (standard error of the mean). Data were analyzed using a one-way ANOVA followed by Dunnett’s multiple comparison tests. *p ≤ .05; **p ≤ .01; ***p ≤ .001; ****p ≤ .0001 versus 6 months, respectively.
