# Supplementary figures and images for: Age-related gene expression signatures from limb skeletal muscles and the diaphragm in mice and rats reveal common and species-specific changes
Source: Skelet Muscle. 2023 Jul 12;13:11. doi: 10.1186/s13395-023-00321-3 (PMC10337157; doi:10.1186/s13395-023-00321-3)

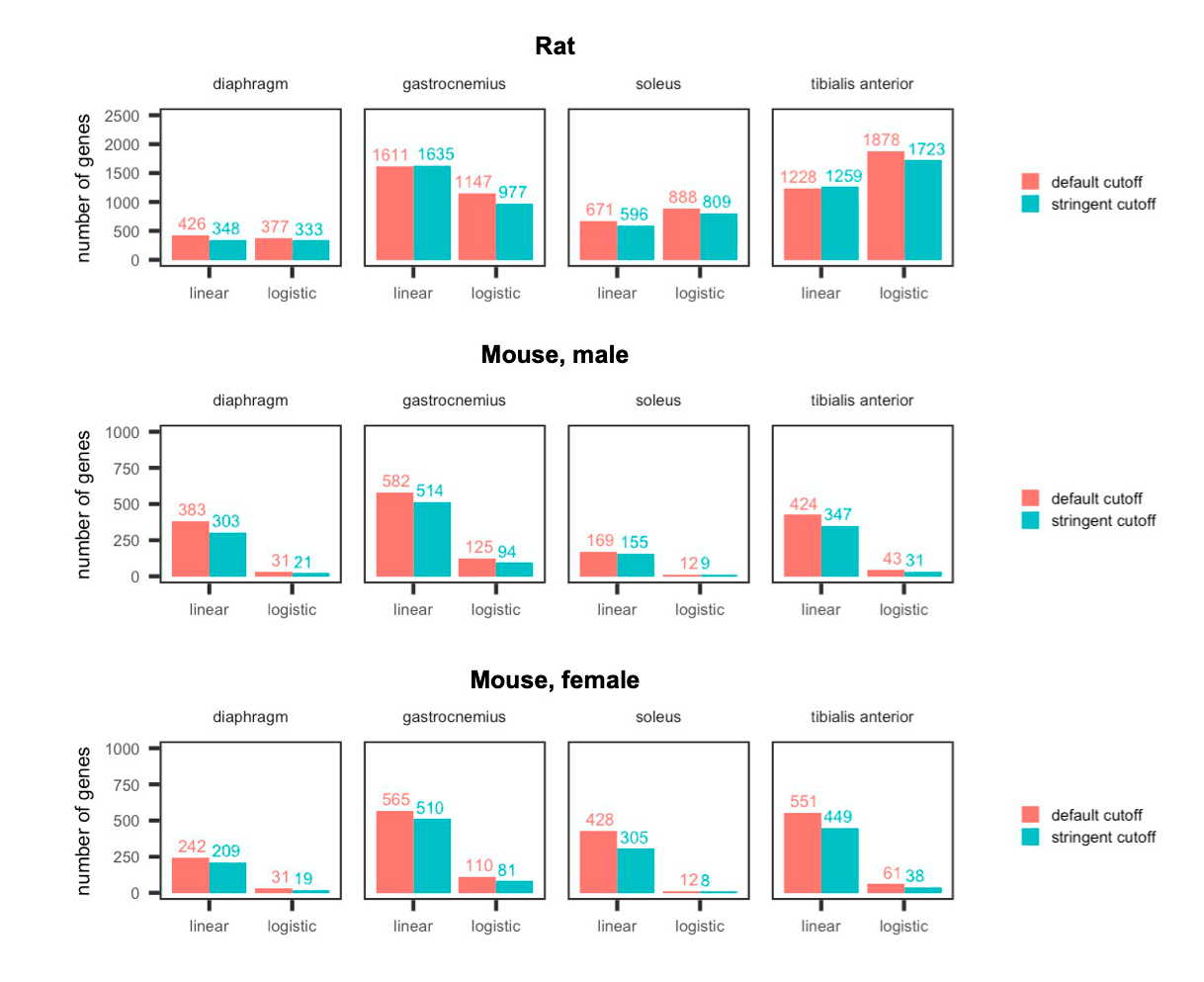

Supplement: Supplementary file 1 — Additional file 1: Table S1. Number of samples for individual muscles in male rats and male and female mice. Number of samples corresponds to the number of animals. E.g. 12 muscles are collected from 12 animals. Table S2. Fold change and adjusted p values of age-related genes in skeletal muscles of rats and mice. Table S3. Probe and primer sequences used for RT-qPCR in mice. Highlighted genes were used as reference genes. Table S4. Probe and primer sequences used for RT-qPCR in rats. Highlighted genes were used as reference genes. Figure S1. Numbers of age-related genes under a stringent cutoff. Figure S2. Gastrocnemius, tibialis anterior and soleus muscle weights in male and female C57Bl6J mice (A, B) and male Sprague Dawley rats (C). Figure S3. Numbers of age-related genes in rat muscles, using lower animal numbers. Figure S4. Numbers of linear and logistic age-related genes in diaphragm, gastrocnemius, soleus and tibialis anterior muscles from female mice. Figure S5. Under stricter examination, rat muscles still enrich for more age-related up-regulated pathways. Figure S6. Age-related genes in male rats and male mice that are associated with immune (A) and mitochondrial (B) pathways. Figure S7. Pathways enriched by age-related genes that were shared between male and female mice. Figure S8. Under stricter examination, rat muscles still enrich for more age-related down-regulated pathways. Figure S9. Transcription factors (TFs) associated with pathways enriched by age-related genes. Figure S10. RT-qPCR validation of top five up- and down-regulated genes in skeletal muscles from male (A and B) and female (C and D) mice. Figure S11. RT-qPCR validation of top five up- and down-regulated genes in skeletal muscles from rats. Figure S12. RT-qPCR validation of transcription factors identified in mice (selected from Figure S9A). Figure S13. RT-qPCR validation of transcription factors identified in rats (selected from Figure S9B). [file 13395_2023_321_MOESM1_ESM.zip › FigS1.jpg]

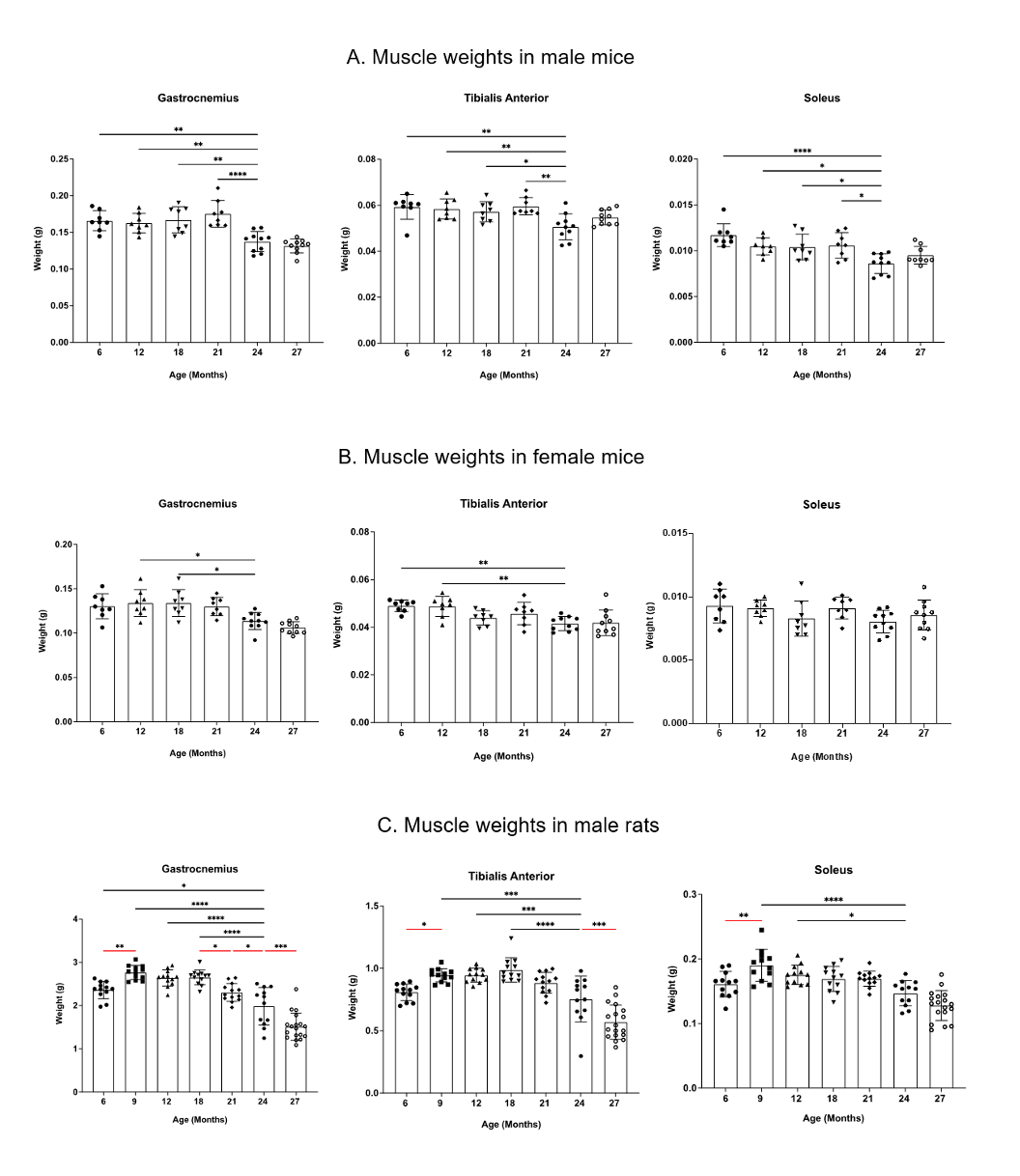

Supplement: Supplementary file 1 — Additional file 1: Table S1. Number of samples for individual muscles in male rats and male and female mice. Number of samples corresponds to the number of animals. E.g. 12 muscles are collected from 12 animals. Table S2. Fold change and adjusted p values of age-related genes in skeletal muscles of rats and mice. Table S3. Probe and primer sequences used for RT-qPCR in mice. Highlighted genes were used as reference genes. Table S4. Probe and primer sequences used for RT-qPCR in rats. Highlighted genes were used as reference genes. Figure S1. Numbers of age-related genes under a stringent cutoff. Figure S2. Gastrocnemius, tibialis anterior and soleus muscle weights in male and female C57Bl6J mice (A, B) and male Sprague Dawley rats (C). Figure S3. Numbers of age-related genes in rat muscles, using lower animal numbers. Figure S4. Numbers of linear and logistic age-related genes in diaphragm, gastrocnemius, soleus and tibialis anterior muscles from female mice. Figure S5. Under stricter examination, rat muscles still enrich for more age-related up-regulated pathways. Figure S6. Age-related genes in male rats and male mice that are associated with immune (A) and mitochondrial (B) pathways. Figure S7. Pathways enriched by age-related genes that were shared between male and female mice. Figure S8. Under stricter examination, rat muscles still enrich for more age-related down-regulated pathways. Figure S9. Transcription factors (TFs) associated with pathways enriched by age-related genes. Figure S10. RT-qPCR validation of top five up- and down-regulated genes in skeletal muscles from male (A and B) and female (C and D) mice. Figure S11. RT-qPCR validation of top five up- and down-regulated genes in skeletal muscles from rats. Figure S12. RT-qPCR validation of transcription factors identified in mice (selected from Figure S9A). Figure S13. RT-qPCR validation of transcription factors identified in rats (selected from Figure S9B). [file 13395_2023_321_MOESM1_ESM.zip › FigS2.jpg]

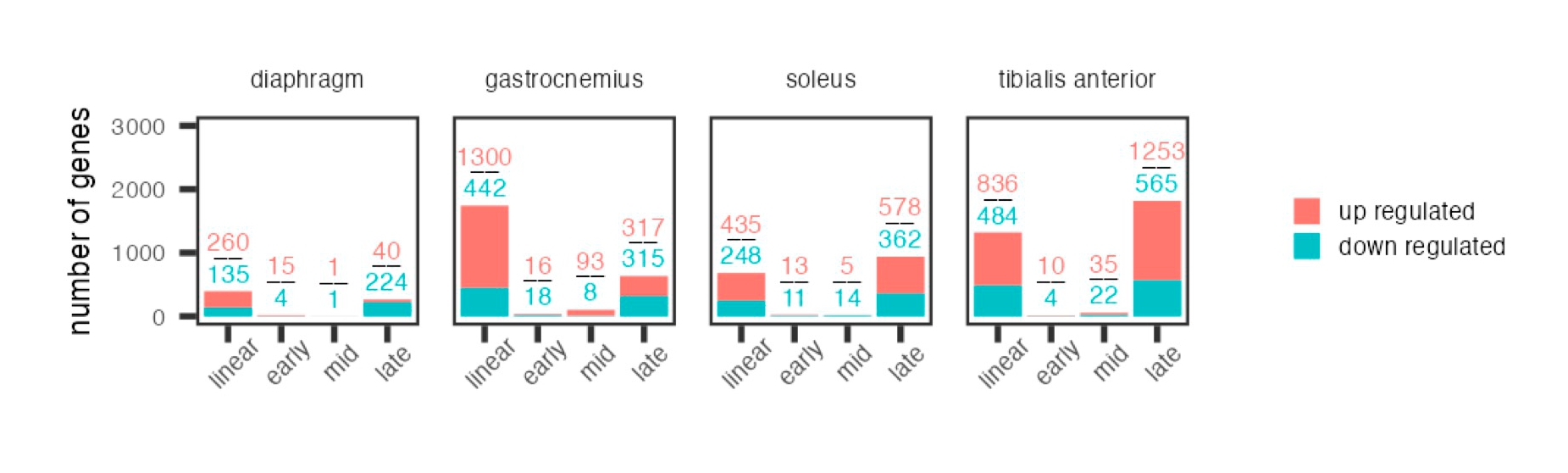

Supplement: Supplementary file 1 — Additional file 1: Table S1. Number of samples for individual muscles in male rats and male and female mice. Number of samples corresponds to the number of animals. E.g. 12 muscles are collected from 12 animals. Table S2. Fold change and adjusted p values of age-related genes in skeletal muscles of rats and mice. Table S3. Probe and primer sequences used for RT-qPCR in mice. Highlighted genes were used as reference genes. Table S4. Probe and primer sequences used for RT-qPCR in rats. Highlighted genes were used as reference genes. Figure S1. Numbers of age-related genes under a stringent cutoff. Figure S2. Gastrocnemius, tibialis anterior and soleus muscle weights in male and female C57Bl6J mice (A, B) and male Sprague Dawley rats (C). Figure S3. Numbers of age-related genes in rat muscles, using lower animal numbers. Figure S4. Numbers of linear and logistic age-related genes in diaphragm, gastrocnemius, soleus and tibialis anterior muscles from female mice. Figure S5. Under stricter examination, rat muscles still enrich for more age-related up-regulated pathways. Figure S6. Age-related genes in male rats and male mice that are associated with immune (A) and mitochondrial (B) pathways. Figure S7. Pathways enriched by age-related genes that were shared between male and female mice. Figure S8. Under stricter examination, rat muscles still enrich for more age-related down-regulated pathways. Figure S9. Transcription factors (TFs) associated with pathways enriched by age-related genes. Figure S10. RT-qPCR validation of top five up- and down-regulated genes in skeletal muscles from male (A and B) and female (C and D) mice. Figure S11. RT-qPCR validation of top five up- and down-regulated genes in skeletal muscles from rats. Figure S12. RT-qPCR validation of transcription factors identified in mice (selected from Figure S9A). Figure S13. RT-qPCR validation of transcription factors identified in rats (selected from Figure S9B). [file 13395_2023_321_MOESM1_ESM.zip › FigS3.jpg]

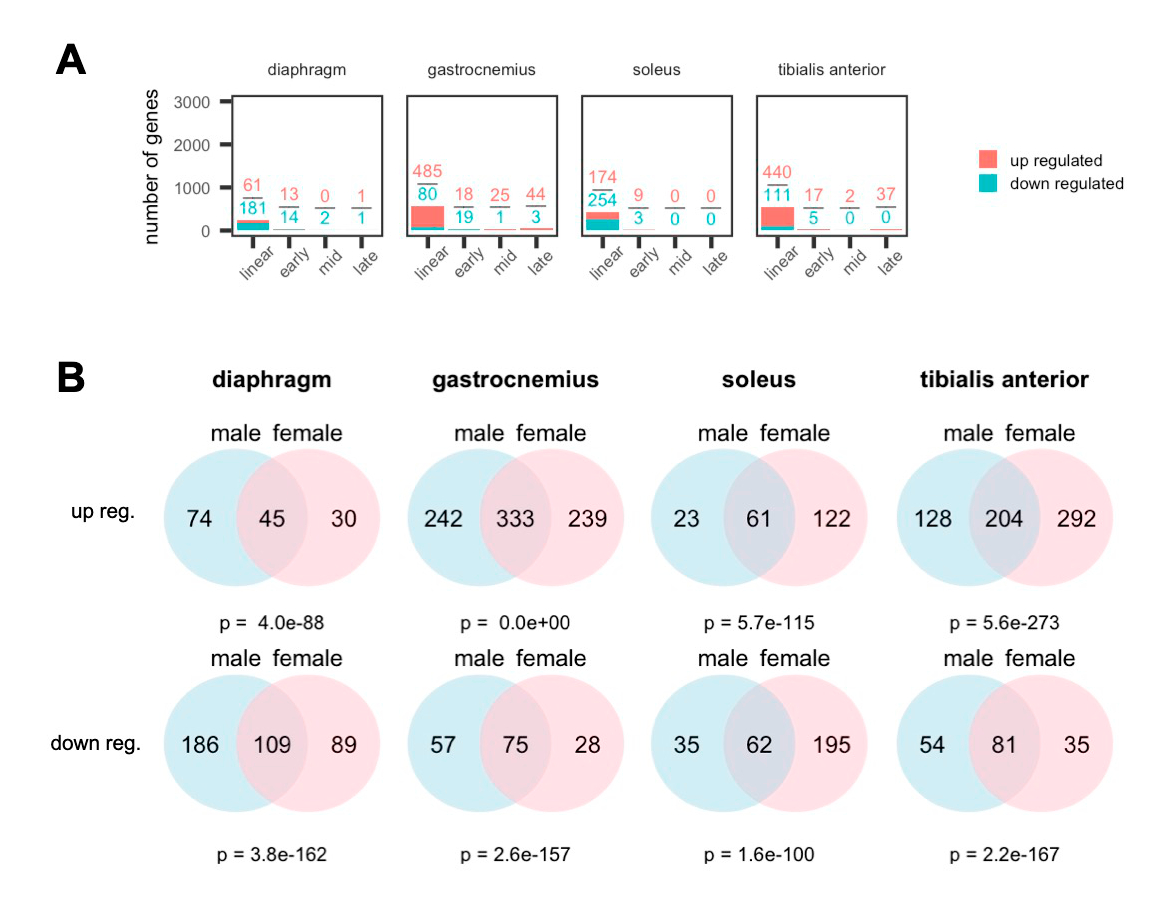

Supplement: Supplementary file 1 — Additional file 1: Table S1. Number of samples for individual muscles in male rats and male and female mice. Number of samples corresponds to the number of animals. E.g. 12 muscles are collected from 12 animals. Table S2. Fold change and adjusted p values of age-related genes in skeletal muscles of rats and mice. Table S3. Probe and primer sequences used for RT-qPCR in mice. Highlighted genes were used as reference genes. Table S4. Probe and primer sequences used for RT-qPCR in rats. Highlighted genes were used as reference genes. Figure S1. Numbers of age-related genes under a stringent cutoff. Figure S2. Gastrocnemius, tibialis anterior and soleus muscle weights in male and female C57Bl6J mice (A, B) and male Sprague Dawley rats (C). Figure S3. Numbers of age-related genes in rat muscles, using lower animal numbers. Figure S4. Numbers of linear and logistic age-related genes in diaphragm, gastrocnemius, soleus and tibialis anterior muscles from female mice. Figure S5. Under stricter examination, rat muscles still enrich for more age-related up-regulated pathways. Figure S6. Age-related genes in male rats and male mice that are associated with immune (A) and mitochondrial (B) pathways. Figure S7. Pathways enriched by age-related genes that were shared between male and female mice. Figure S8. Under stricter examination, rat muscles still enrich for more age-related down-regulated pathways. Figure S9. Transcription factors (TFs) associated with pathways enriched by age-related genes. Figure S10. RT-qPCR validation of top five up- and down-regulated genes in skeletal muscles from male (A and B) and female (C and D) mice. Figure S11. RT-qPCR validation of top five up- and down-regulated genes in skeletal muscles from rats. Figure S12. RT-qPCR validation of transcription factors identified in mice (selected from Figure S9A). Figure S13. RT-qPCR validation of transcription factors identified in rats (selected from Figure S9B). [file 13395_2023_321_MOESM1_ESM.zip › FigS4.jpg]

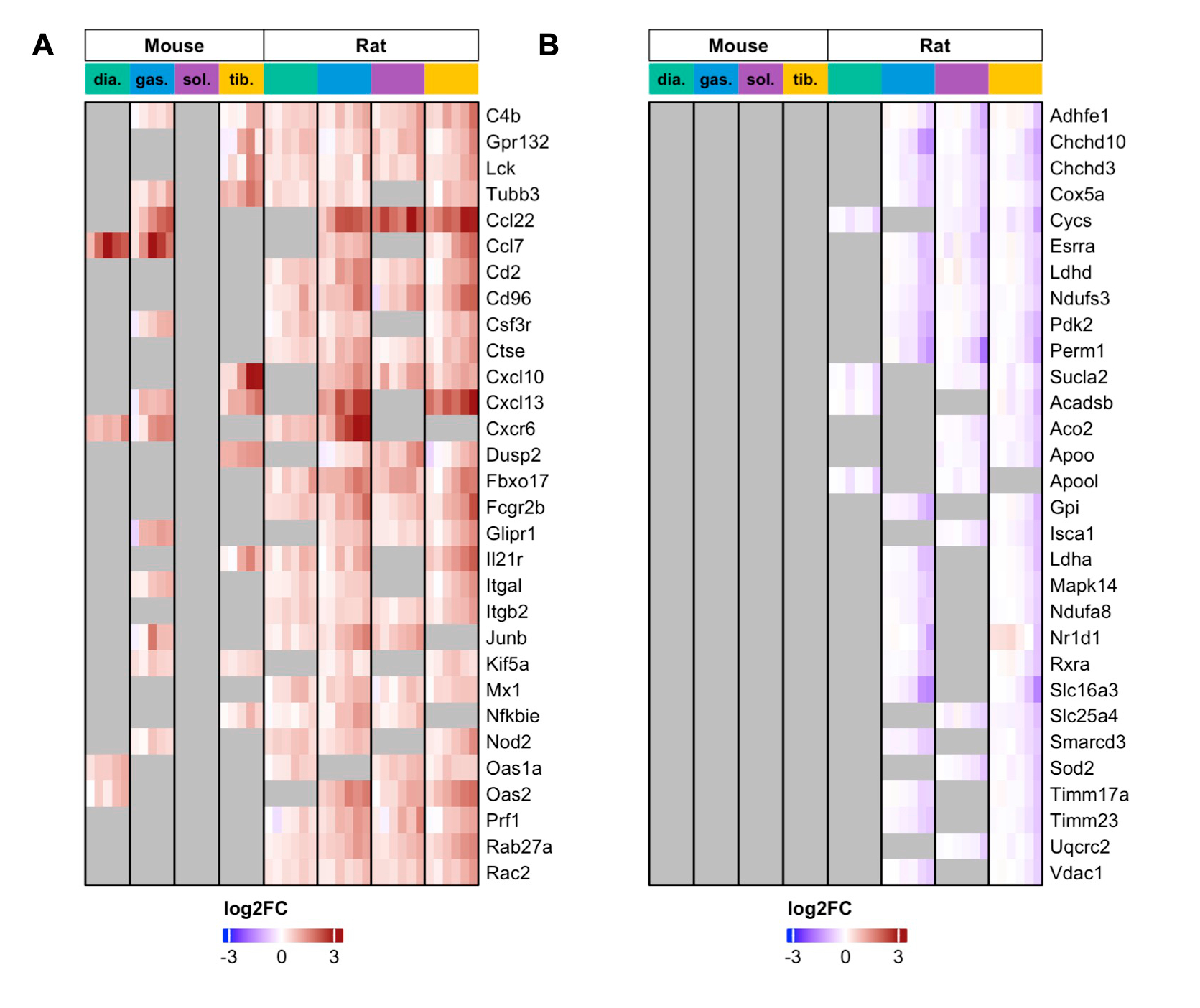

Supplement: Supplementary file 1 — Additional file 1: Table S1. Number of samples for individual muscles in male rats and male and female mice. Number of samples corresponds to the number of animals. E.g. 12 muscles are collected from 12 animals. Table S2. Fold change and adjusted p values of age-related genes in skeletal muscles of rats and mice. Table S3. Probe and primer sequences used for RT-qPCR in mice. Highlighted genes were used as reference genes. Table S4. Probe and primer sequences used for RT-qPCR in rats. Highlighted genes were used as reference genes. Figure S1. Numbers of age-related genes under a stringent cutoff. Figure S2. Gastrocnemius, tibialis anterior and soleus muscle weights in male and female C57Bl6J mice (A, B) and male Sprague Dawley rats (C). Figure S3. Numbers of age-related genes in rat muscles, using lower animal numbers. Figure S4. Numbers of linear and logistic age-related genes in diaphragm, gastrocnemius, soleus and tibialis anterior muscles from female mice. Figure S5. Under stricter examination, rat muscles still enrich for more age-related up-regulated pathways. Figure S6. Age-related genes in male rats and male mice that are associated with immune (A) and mitochondrial (B) pathways. Figure S7. Pathways enriched by age-related genes that were shared between male and female mice. Figure S8. Under stricter examination, rat muscles still enrich for more age-related down-regulated pathways. Figure S9. Transcription factors (TFs) associated with pathways enriched by age-related genes. Figure S10. RT-qPCR validation of top five up- and down-regulated genes in skeletal muscles from male (A and B) and female (C and D) mice. Figure S11. RT-qPCR validation of top five up- and down-regulated genes in skeletal muscles from rats. Figure S12. RT-qPCR validation of transcription factors identified in mice (selected from Figure S9A). Figure S13. RT-qPCR validation of transcription factors identified in rats (selected from Figure S9B). [file 13395_2023_321_MOESM1_ESM.zip › FigS6.jpg]

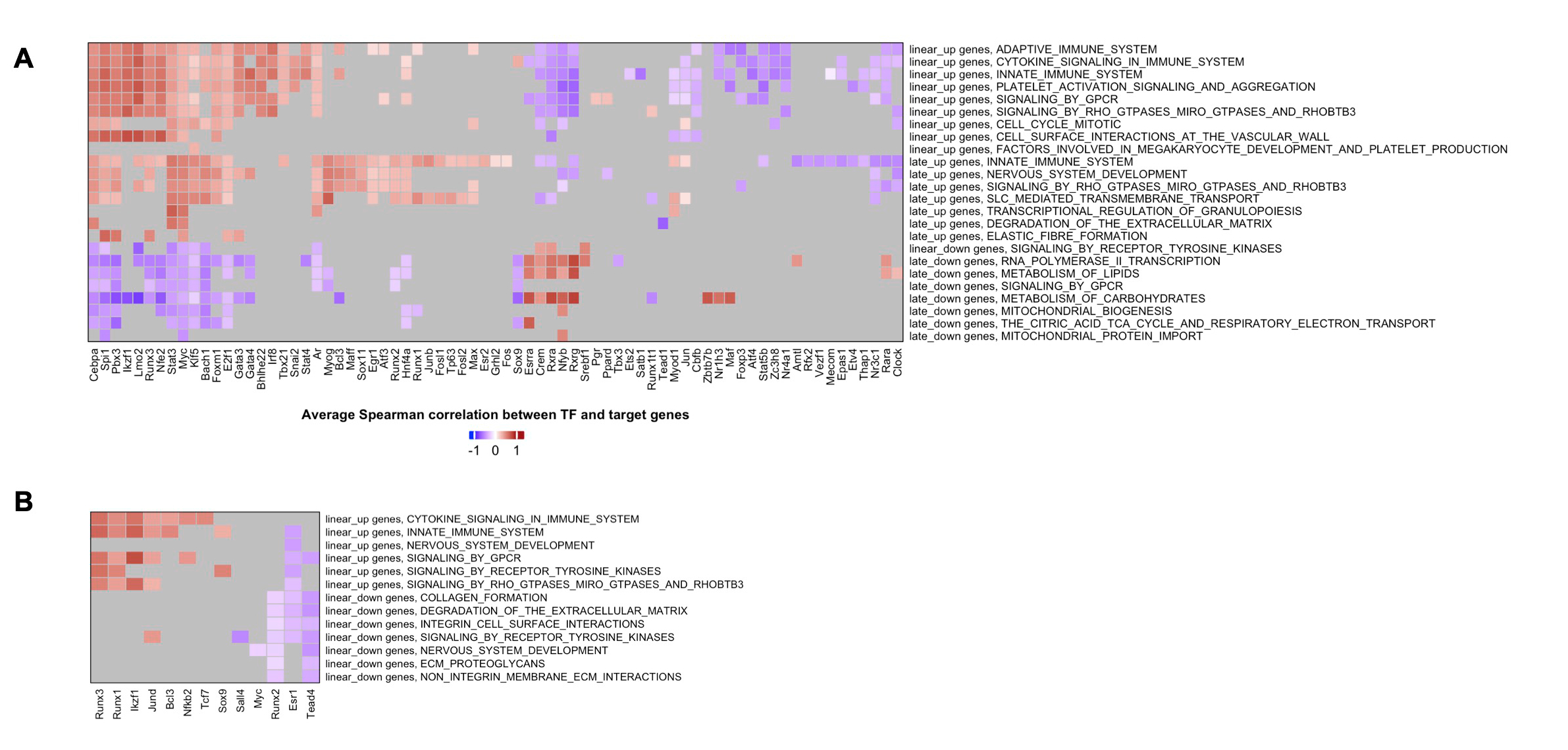

Supplement: Supplementary file 1 — Additional file 1: Table S1. Number of samples for individual muscles in male rats and male and female mice. Number of samples corresponds to the number of animals. E.g. 12 muscles are collected from 12 animals. Table S2. Fold change and adjusted p values of age-related genes in skeletal muscles of rats and mice. Table S3. Probe and primer sequences used for RT-qPCR in mice. Highlighted genes were used as reference genes. Table S4. Probe and primer sequences used for RT-qPCR in rats. Highlighted genes were used as reference genes. Figure S1. Numbers of age-related genes under a stringent cutoff. Figure S2. Gastrocnemius, tibialis anterior and soleus muscle weights in male and female C57Bl6J mice (A, B) and male Sprague Dawley rats (C). Figure S3. Numbers of age-related genes in rat muscles, using lower animal numbers. Figure S4. Numbers of linear and logistic age-related genes in diaphragm, gastrocnemius, soleus and tibialis anterior muscles from female mice. Figure S5. Under stricter examination, rat muscles still enrich for more age-related up-regulated pathways. Figure S6. Age-related genes in male rats and male mice that are associated with immune (A) and mitochondrial (B) pathways. Figure S7. Pathways enriched by age-related genes that were shared between male and female mice. Figure S8. Under stricter examination, rat muscles still enrich for more age-related down-regulated pathways. Figure S9. Transcription factors (TFs) associated with pathways enriched by age-related genes. Figure S10. RT-qPCR validation of top five up- and down-regulated genes in skeletal muscles from male (A and B) and female (C and D) mice. Figure S11. RT-qPCR validation of top five up- and down-regulated genes in skeletal muscles from rats. Figure S12. RT-qPCR validation of transcription factors identified in mice (selected from Figure S9A). Figure S13. RT-qPCR validation of transcription factors identified in rats (selected from Figure S9B). [file 13395_2023_321_MOESM1_ESM.zip › FigS9.jpg]

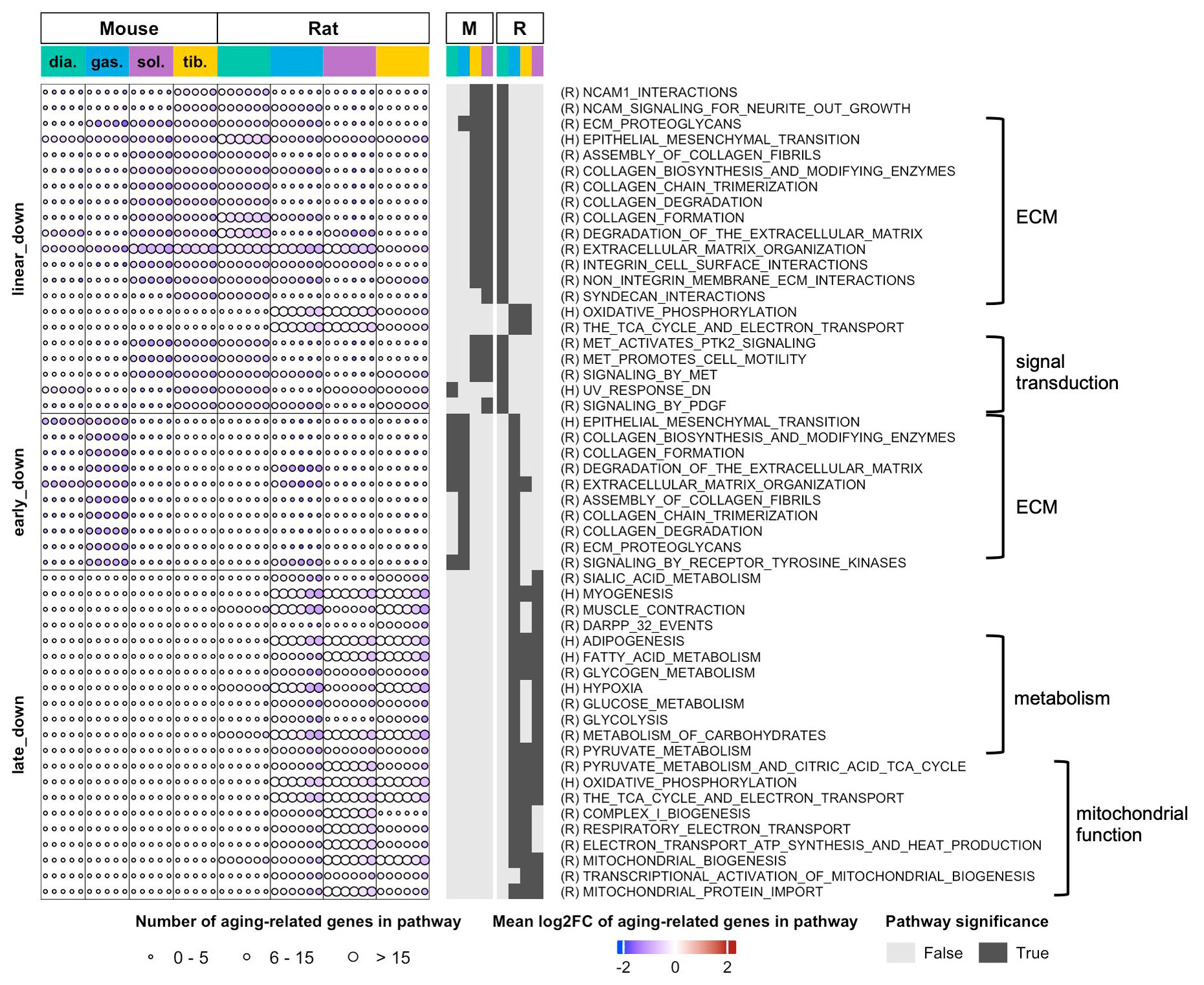

Supplement: Supplementary file 1 — Additional file 1: Table S1. Number of samples for individual muscles in male rats and male and female mice. Number of samples corresponds to the number of animals. E.g. 12 muscles are collected from 12 animals. Table S2. Fold change and adjusted p values of age-related genes in skeletal muscles of rats and mice. Table S3. Probe and primer sequences used for RT-qPCR in mice. Highlighted genes were used as reference genes. Table S4. Probe and primer sequences used for RT-qPCR in rats. Highlighted genes were used as reference genes. Figure S1. Numbers of age-related genes under a stringent cutoff. Figure S2. Gastrocnemius, tibialis anterior and soleus muscle weights in male and female C57Bl6J mice (A, B) and male Sprague Dawley rats (C). Figure S3. Numbers of age-related genes in rat muscles, using lower animal numbers. Figure S4. Numbers of linear and logistic age-related genes in diaphragm, gastrocnemius, soleus and tibialis anterior muscles from female mice. Figure S5. Under stricter examination, rat muscles still enrich for more age-related up-regulated pathways. Figure S6. Age-related genes in male rats and male mice that are associated with immune (A) and mitochondrial (B) pathways. Figure S7. Pathways enriched by age-related genes that were shared between male and female mice. Figure S8. Under stricter examination, rat muscles still enrich for more age-related down-regulated pathways. Figure S9. Transcription factors (TFs) associated with pathways enriched by age-related genes. Figure S10. RT-qPCR validation of top five up- and down-regulated genes in skeletal muscles from male (A and B) and female (C and D) mice. Figure S11. RT-qPCR validation of top five up- and down-regulated genes in skeletal muscles from rats. Figure S12. RT-qPCR validation of transcription factors identified in mice (selected from Figure S9A). Figure S13. RT-qPCR validation of transcription factors identified in rats (selected from Figure S9B). [file 13395_2023_321_MOESM1_ESM.zip › FigS8.jpg]

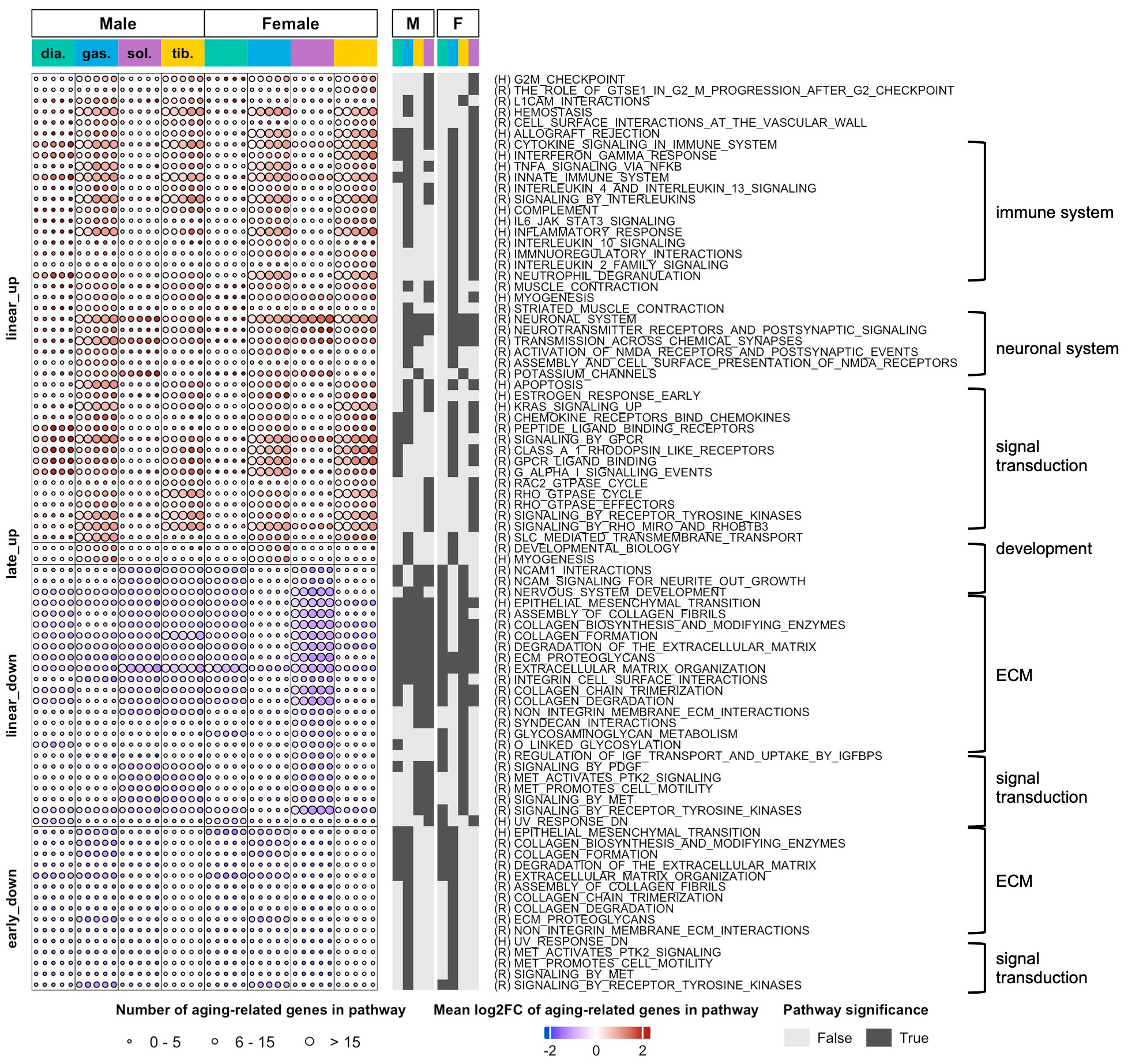

Supplement: Supplementary file 1 — Additional file 1: Table S1. Number of samples for individual muscles in male rats and male and female mice. Number of samples corresponds to the number of animals. E.g. 12 muscles are collected from 12 animals. Table S2. Fold change and adjusted p values of age-related genes in skeletal muscles of rats and mice. Table S3. Probe and primer sequences used for RT-qPCR in mice. Highlighted genes were used as reference genes. Table S4. Probe and primer sequences used for RT-qPCR in rats. Highlighted genes were used as reference genes. Figure S1. Numbers of age-related genes under a stringent cutoff. Figure S2. Gastrocnemius, tibialis anterior and soleus muscle weights in male and female C57Bl6J mice (A, B) and male Sprague Dawley rats (C). Figure S3. Numbers of age-related genes in rat muscles, using lower animal numbers. Figure S4. Numbers of linear and logistic age-related genes in diaphragm, gastrocnemius, soleus and tibialis anterior muscles from female mice. Figure S5. Under stricter examination, rat muscles still enrich for more age-related up-regulated pathways. Figure S6. Age-related genes in male rats and male mice that are associated with immune (A) and mitochondrial (B) pathways. Figure S7. Pathways enriched by age-related genes that were shared between male and female mice. Figure S8. Under stricter examination, rat muscles still enrich for more age-related down-regulated pathways. Figure S9. Transcription factors (TFs) associated with pathways enriched by age-related genes. Figure S10. RT-qPCR validation of top five up- and down-regulated genes in skeletal muscles from male (A and B) and female (C and D) mice. Figure S11. RT-qPCR validation of top five up- and down-regulated genes in skeletal muscles from rats. Figure S12. RT-qPCR validation of transcription factors identified in mice (selected from Figure S9A). Figure S13. RT-qPCR validation of transcription factors identified in rats (selected from Figure S9B). [file 13395_2023_321_MOESM1_ESM.zip › FigS7.jpg]

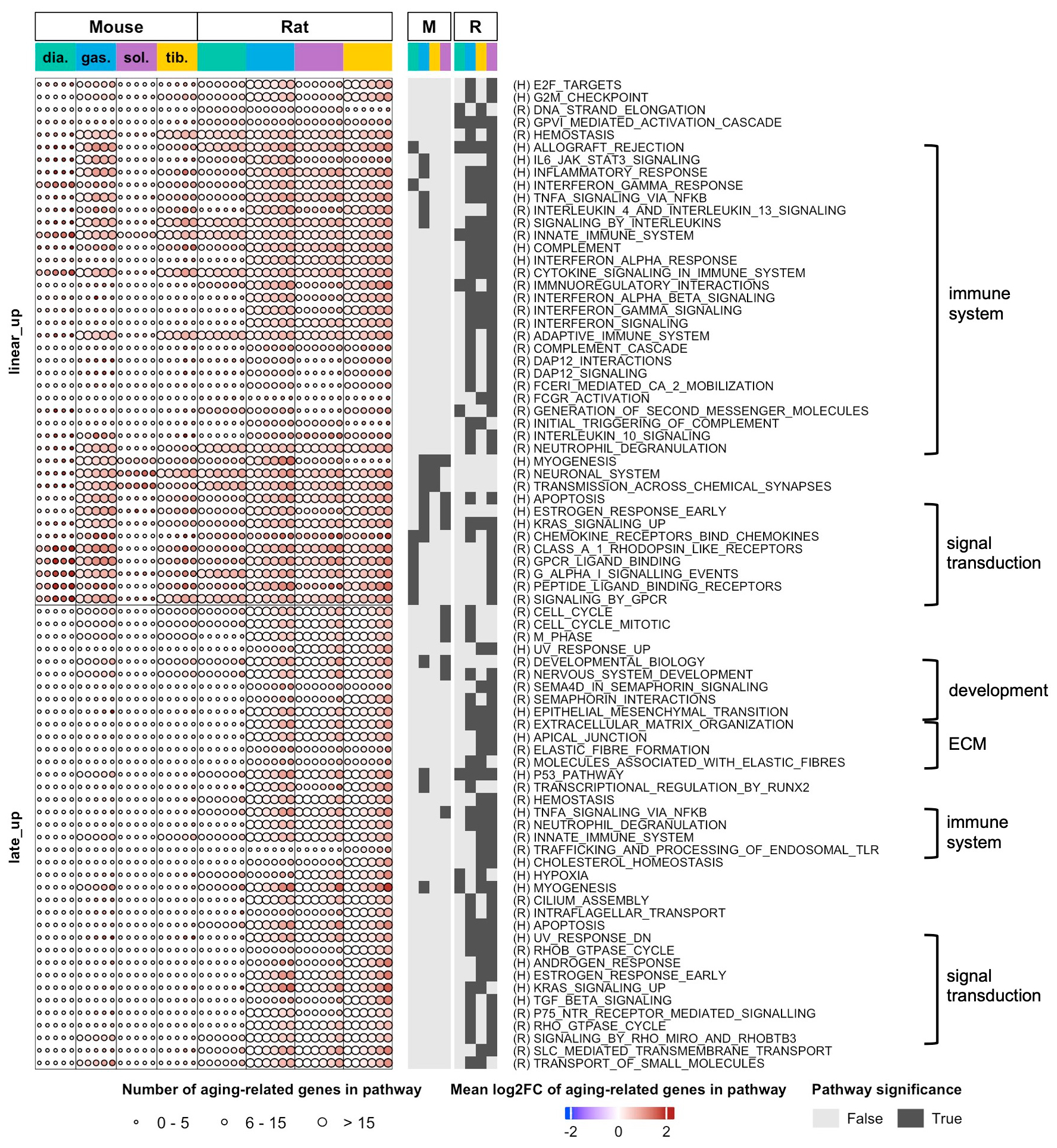

Supplement: Supplementary file 1 — Additional file 1: Table S1. Number of samples for individual muscles in male rats and male and female mice. Number of samples corresponds to the number of animals. E.g. 12 muscles are collected from 12 animals. Table S2. Fold change and adjusted p values of age-related genes in skeletal muscles of rats and mice. Table S3. Probe and primer sequences used for RT-qPCR in mice. Highlighted genes were used as reference genes. Table S4. Probe and primer sequences used for RT-qPCR in rats. Highlighted genes were used as reference genes. Figure S1. Numbers of age-related genes under a stringent cutoff. Figure S2. Gastrocnemius, tibialis anterior and soleus muscle weights in male and female C57Bl6J mice (A, B) and male Sprague Dawley rats (C). Figure S3. Numbers of age-related genes in rat muscles, using lower animal numbers. Figure S4. Numbers of linear and logistic age-related genes in diaphragm, gastrocnemius, soleus and tibialis anterior muscles from female mice. Figure S5. Under stricter examination, rat muscles still enrich for more age-related up-regulated pathways. Figure S6. Age-related genes in male rats and male mice that are associated with immune (A) and mitochondrial (B) pathways. Figure S7. Pathways enriched by age-related genes that were shared between male and female mice. Figure S8. Under stricter examination, rat muscles still enrich for more age-related down-regulated pathways. Figure S9. Transcription factors (TFs) associated with pathways enriched by age-related genes. Figure S10. RT-qPCR validation of top five up- and down-regulated genes in skeletal muscles from male (A and B) and female (C and D) mice. Figure S11. RT-qPCR validation of top five up- and down-regulated genes in skeletal muscles from rats. Figure S12. RT-qPCR validation of transcription factors identified in mice (selected from Figure S9A). Figure S13. RT-qPCR validation of transcription factors identified in rats (selected from Figure S9B). [file 13395_2023_321_MOESM1_ESM.zip › FigS5.jpg]
